# Direct localization of detergents and bacteriorhodopsin in the lipidic cubic phase by small-angle neutron scattering

**DOI:** 10.1107/S2052252520013974

**Published:** 2021-01-01

**Authors:** Thomas Cleveland IV, Emily Blick, Susan Krueger, Anna Leung, Tamim Darwish, Paul Butler

**Affiliations:** a National Institute of Standards and Technology and Institute for Bioscience and Biotechnology Research, 9600 Gudelsky Drive, Rockville, MD 20850, USA; b National Institute of Standards and Technology Center for Neutron Research, 100 Bureau Drive, Gaithersburg, MD 20899, USA; c National Deuteration Facility, Australian Nuclear Science and Technology Organisation, Locked Bag 2001, Kirrawee DC, NSW 2232, Australia; d Scientific Activities Division, European Spallation Source ERIC, Lund 224 84, Sweden; e Department of Chemistry, University of Tennessee, 552 Buehler Hall, 1420 Circle Dr., Knoxville, TN 37996-1600, USA; f Department of Chemical and Biomolecular Engineering, University of Delaware, 150 Academy Street, Colburn Laboratory, Newark, DE 19716, USA

**Keywords:** membrane proteins, protein structure, SANS, solution scattering, structural biology, crystallization, crystal growth, neutron crystallography

## Abstract

Lipidic cubic phase (LCP) crystallization methods have been essential in obtaining crystals of certain membrane proteins, particularly G-protein-coupled receptors. LCP crystallization is generally optimized across a large number of potential variables, one of which may be the choice of the solubilizing detergent. A better fundamental understanding of the behavior of detergents in the LCP may guide and simplify the detergent selection process. This work investigates the distribution of protein and detergent in LCP using the membrane protein bacteriorhodopsin (bR), with the LCP prepared from highly deuterated monoolein to allow contrast-matched small-angle neutron scattering. Contrast-matching allows the scattering from the LCP bilayer itself to be suppressed, so that the distribution and behavior of the protein and detergent can be directly studied. The results showed that, for several common detergents, the detergent micelle dissociates and incorporates into the LCP bilayer essentially as free detergent monomers. In addition, the detergent octyl glucoside dissociates from bR, and neither the protein nor detergent forms clusters in the LCP. The lack of detergent assemblies in the LCP implies that, upon incorporation, micelle sizes and protein/detergent interactions become less important than they would be in solution crystallization. Crystallization screening confirmed this idea, with crystals obtained from bR in the presence of most detergents tested. Thus, in LCP crystallization, detergents can be selected primarily on the basis of protein stabilization in solution, with crystallization suitability a lesser consideration.

## Abstract 2

Using small-angle neutron scattering with contrast-matching of the lipidic cubic phase (LCP) bilayer, we determined that bacteriorhodopsin (bR) and detergents are largely dissociated upon incorporation into the LCP. This implies that the crystallization of membrane proteins in LCP is relatively insensitive to the choice of detergent, unlike the solution case, and this was experimentally verified with several detergents for bR.

## 1. Introduction   

Incorporation of membrane proteins into lipidic mesophases such as the lipidic cubic phase (LCP) has become an important method for obtaining crystals for structural studies. This procedure is often referred to as *‘in meso’* crystallization, in contrast with *‘in surfo’*, which refers to direct solution crystallization of the protein/detergent complex. Although proteins of many types have proven amenable to *in meso* crystallization (alpha helical, beta barrel and a range of protein sizes) (Caffrey, 2015 ▸), the technique has been particularly useful for obtaining crystals of G-protein-coupled receptors (GPCRs) (Grisshammer, 2017 ▸). A recent search of the PDB, using the ‘Membrane Protein Browser’ and specifying GPCRs, returned 420 entries for GPCRs solved by X-ray crystallography, of which 236 cited some variation of *in meso* as the crystallization technique (compared with 86 that listed vapor diffusion, most of which were likely *in surfo*, and 97 that did not list a method). Presumably, one reason *in surfo* crystallization may fail for these proteins is that they are comparatively small, and often lack large extramembrane domains for crystal packing, while the transmembrane region is occluded by the detergent micelle. When membrane proteins are crystallized from detergent solution, a belt of detergent similar to a micelle remains bound around the hydrophobic region of the membrane protein, and must be accommodated by the crystal lattice. This detergent belt has been directly visualized using neutron diffraction (Prince *et al.*, 2003 ▸). The size of the bound micelle varies with detergent, and can be large enough to encroach on the polar regions of the protein (which are needed to form crystal contacts) (Bamber *et al.*, 2006 ▸). Proteins with small polar regions relative to the transmembrane region, such as GPCRs, would be be more affected by this problem. By contrast, upon incorporation into the LCP, the micelle is generally assumed to dissociate (Caffrey, 2000 ▸), allowing crystal contacts and packing to be mediated by transmembrane regions of the protein as well. A convincing example of this observation can be found in the work by Cherezov *et al.* (2006 ▸), where crystals of the light-harvesting II complex grown *in surfo* require space in the lattice for the detergent belt around the transmembrane region; whereas *in meso* crystals of the same protein allow for close packing between transmembrane regions of adjacent proteins. A recent review that discusses these ideas is available (Birch *et al.*, 2018 ▸).

A common presumption is that the membrane protein and detergent fully dissociate upon incorporation into LCP (Caffrey, 2000 ▸), but there has been little direct experimental evidence for this. In addition, if this hypothesis is true, it raises the possibility that the choice of detergent may not be of great importance to *in meso* crystallization at all. There would be significant benefit from such a simplification, since the number of variables needing to be screened for crystallizing membrane proteins is ordinarily very high; in addition to the usual screening of precipitants, cofactors, temperature, *etc*. membrane proteins also usually require optimization of the detergent, and (for *in meso* crystallization) the lipid and additives such as cholesterol. Furthermore, detergents that are optimal for protein stability in solution are not necessarily optimal for *in surfo* crystallization. Several examples of membrane proteins that were soluble in multiple detergents, but where crystallization success was exquisitely sensitive to small structural variations in the detergent, are discussed in the work by Ostermeier & Michel (1997 ▸). Among these was a cytochrome *c* oxidase that gave crystals diffracting to 8 Å in dodecyl maltoside, 2.6 Å in undecyl maltoside and no crystals at all in decyl maltoside. Short-chain detergents are desirable for obtaining GPCR crystals, but are sometimes too denaturing and require stabilizing mutations to be introduced into the protein. Long-chain detergents are thought to be more stabilizing, but may require the use of antibody fragments or the addition of soluble domains through protein engineering to overcome occlusion by the large bound micelle (Grisshammer, 2017 ▸). If detergents could instead be selected for solution stability only, without needing to consider their suitability for crystallization, this would represent an important advantage to LCP crystallization.

With this question in mind, we have used small-angle neutron scattering (SANS) to study the distribution and behavior of membrane proteins and detergent after incorporation into the LCP. SANS has proven to be a powerful technique for studying the statistical average bulk structures in materials, elucidating size, shapes, orientations, distributions and interactions (Guinier & Fournet, 1947 ▸; Glatter & Kratky, 1982 ▸; Higgins & Benoît, 1996 ▸). In this work we take advantage of the fact that the scattering power of neutrons (closely related to the index of refraction) can vary a great deal between isotopes of the same element. In particular, the scattering power for hydrogen is vastly different than for deuterium. This makes SANS especially useful in soft matter studies, where the use of deuterated versus hydrogenated materials and/or solvents can significantly reduce the complexity of the system by effectively masking out selected portions of the material, a technique often referred to as contrast-matching.

**Figure 1:**
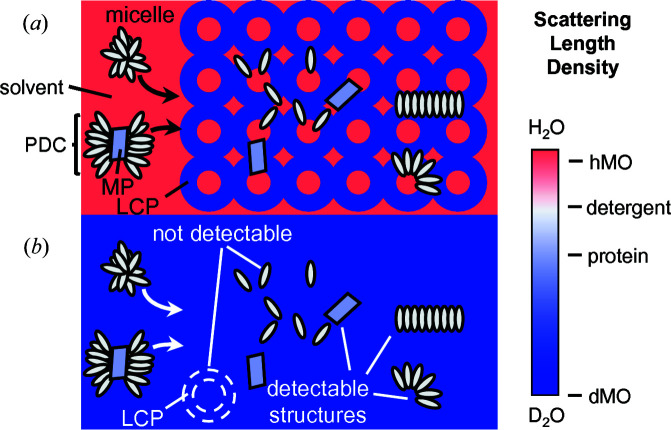
Use of contrast-matched LCP in SANS. The cartoon depicts conditions under which the lipid (*a*) is not contrast-matched and (*b*) is contrast-matched. Contrast-matching eliminates most of the scattering from the LCP, allowing any structures formed by non-deuterated components of the system to be observed by SANS. These could potentially include membrane protein (MP) molecules, protein/detergent complexes (PDCs) or detergent micelles/aggregates. Detergent monomers are not detectable due to their small size.

For crystallization studies, we selected bacteriorhodopsin (bR), which is the prototypical model system for GPCR-like protein incorporation into the LCP, along with several common detergents. Highly deuterated monoolein (dMO) was used to form the LCP as it is mostly contrast-matched by pure D_2_O, minimizing the scattering from the lipid phase. This is critical since the lipid constitutes roughly 60% of the mass of the sample, whereas the protein and detergent together are around 5% at most. Thus, in the contrast-matched LCP, direct measurement of scattering from small amounts of embedded protein and detergent is possible (Fig. 1 ▸). In our studies, we examined the behavior of different detergents, as well as bR, before and after incorporation into the LCP. Finally, we attempted crystallization trials with bR in these detergents in order to relate the observed scattering behavior to crystallization.

## 2. Results   

### 2.1. Scattering from detergent solution micelles   

We initially measured SANS from D_2_O micellar solutions for a panel of 32 detergents (Fig. S1 and Table S1 of the supporting information) taken from common classes used in membrane protein biochemical and crystallization studies. Scattering from a suspension of homogeneous particles in solution (*e.g.* protein molecules or micelles) or any pseudo two-component system, where the center of mass scattering can be separated from the ‘particle’ scattering, can generally be written as
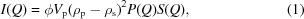
where *Q*, the momentum transfer, is given as 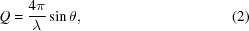
where λ is the wavelength of the neutron (or X-ray or light); 2θ is the scattering angle; ϕ is the total volume fraction of particles; *V*_p_ is the volume of one particle; ρ_p_ and ρ_s_ are the scattering length densities (SLDs, representing the scattering power of each component) of the particle and solvent, respectively; and *P*(*Q*) and *S*(*Q*) are the *Q*-dependent parts of the scattering which contain structural information about the sample (Feigin & Svergun, 1987 ▸). *P*(*Q*), the particle form factor, contains the shape-dependent scattering information and *S*(*Q*), the structure factor, contains the inter-particle scattering information (or correlations), and is essentially the Fourier transform of the pair correlation function *g*(*r*). Note that for sufficiently dilute solutions there are no inter-particle correlations and *S*(*Q*) = 1.

Datasets were fit to a solid ellipsoid of revolution *P*(*Q*) (Feigin & Svergun, 1987 ▸) to determine the approximate size and shape of the micelles. A ‘hard sphere’ *S*(*Q*) (Percus & Yevick, 1958 ▸; Kotlarchyk & Chen, 1983 ▸) was also included in the model. The results are tabulated in Table S1, with fitting details discussed in Fig. S2. A subset of the detergents was then selected for incorporation into the LCP for SANS measurements, to measure any micelle dissociation and possible formation of other structures. The detergents chosen were Anapoe X-100 (AX-100), *n*-dodecyl-β-d-maltopyranoside (DDM), *n*-octyl-β-d-glucoside (OG), lauryl maltose neopentyl glycol (LMNG), nonaethylene glycol monododecyl ether (C_12_E_9_) and *N*,*N*-dimethyldodecylamine-*N*-oxide (LDAO). In selecting these, we intended to sample detergents from different classes, and with different micelle dimensions (a property indicative of the curvature-inducing effect of the detergent and thus its potential effects on the cubic phase). The selected detergents had micelles with (equatorial) diameters ranging from 2.6 to 5.0 nm, and (polar) lengths ranging from 6.6 to 21.4 nm (with the exception of LMNG, which formed very extended rod-like structures of 3.7 nm diameter, and length greater than the measurement limit of about 100 nm). Selection was also based in part on the practical significance of the detergent (*i.e.* those commonly used in membrane protein biochemical and structural studies).

### 2.2. Preparation of contrast-matched lipidic cubic phase   

**Figure 2:**
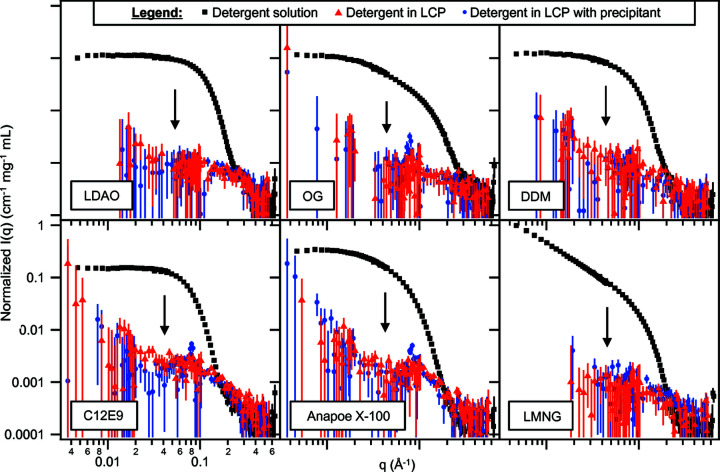
Distribution and aggregation state of detergents in LCP. SANS curves were compared for detergents in solution, after incorporation into LCP, and after the addition of 2 mol l^−1^ phosphate precipitant to the LCP. Solution micelle scattering clearly disappears (see arrows) upon detergent incorporation into LCP. SANS curves are incoherent background-subtracted. Scattering from ‘blank’ LCP has been subtracted from the scattering of LCP with detergent (all other components of the blank, including precipitant, were at the same concentration). Intensities were normalized by the detergent concentration. Due to the high scattering of LCP at low *q* (see discussion in Section 4.3 and Fig. S5), the subtraction procedure becomes unreliable at *q* 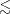 0.02 Å^−1^, but this is well separated from the range of interest for the detergent micellar scattering, which is around 0.02 Å^−1^ < *q* < 0.2 Å^−1^. Unless otherwise stated, error bars in scattering curves are plus or minus one standard deviation and represent the uncertainty associated with counting statistics.

LCP was prepared from dMO in 100% D_2_O buffer and measured by SANS [Fig. S3(*a*), blue]. A small residual Bragg peak was observed near 0.8 Å^−1^ due to the imperfect contrast-matching between dMO head and tail groups, but scattering was reduced by two orders of magnitude compared with non-contrast-matched LCP [Fig. S3(*a*), light red]. A better match would be possible by preparing a mixture including both non-deuterated monoolein (hMO) and dMO like in the work by van’t Hag *et al.*, (2019 ▸), or even better, though much more expensive, a mixture of dMO with MO containing deuterated heads and protonated tails. We chose not to do so for several reasons, but primarily because we found that the small remaining signal from LCP could be sufficiently well subtracted so as not to interfere with data interpretation. Such mixtures also cannot be separated, precluding the re-use of the very expensive fully deuterated dMO and thus dramatically reducing the number of possible experiments. Other reasons include the fact that the mixtures would slightly increase the flat incoherent background, and the probability that ^1^H-detergents, once dispersed into the LCP, would slightly alter the contrast between the heads and tails (as indeed is seen in Fig. 2 ▸ and S4) resulting in the appearance of a residual Bragg peak in any case.

The steep upturn at very low *Q* seen in Fig. S3(*a*) can be attributed to the inevitable uptake, and subsequent micronization, of air into the sample by the required syringe-mixing phase of the sample preparation, yielding a large number of microbubbles of air (see Section 4.3 and Fig. S5 for additional information).

### 2.3. Distribution and aggregation state of detergent incorporated into the LCP   

Detergent solutions (3% *w*/*v*) in D_2_O were mixed with dMO to prepare LCP containing 20 mg of detergent per millilitre of monoolein, and SANS measurements were performed. Scattering from ‘blank’ LCP (prepared in the same way but without detergent) was subtracted from the LCP/detergent scattering (Fig. 2 ▸) and compared with scattering from the solution micelles (black squares) after normalization by detergent concentration. LCP scattering curves without subtraction or normalization are shown in Fig. S4(*a*). Scattering from the solution micelles disappeared upon incorporation into the LCP, indicating a complete loss of detergent aggregation. We also note that, as suggested above, the incorporated detergents slightly alter the intensity of the residual Bragg peak [Fig. S4(*a*), blue curves]. This would be expected if the detergent partitions into the lipid bilayer, where it would add differing amounts of contrast to the headgroup versus  the tail layers of the bilayer. This is especially noticeable in the case of LDAO, which causes the Bragg peak to disappear. LDAO has the lowest calculated SLD (Table S2) of the chosen detergents, while the deuterated tail of dMO has an SLD slightly higher than that of D_2_O; therefore, LDAO may pull the average SLD of the tail layer close enough to the match condition to cause the Bragg peak to disappear. This further supports that the detergents are incorporated as co-amphiphiles into the lipidic bilayer upon mixing.

The same contrast-matched LCP/detergent samples were also examined using small-angle X-ray scattering (SAXS), where the lipids are not contrast-matched and their scattering would thus be expected to dominate any signal from the detergent (Fig. S6). SAXS confirmed the formation of the expected 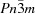 LCP and allowed indexing for lattice parameter determination. The lattice parameter varied as a function of detergent identity from 10.1 to 11.3 nm, presumably due to the different detergent monomers’ effect on the preferred curvature of the LCP.

Finally, we repeated the SANS measurements after adding 2 mol l^−1^ sodium phosphate precipitant (similar to what would be used in bR crystallization) to the LCP/detergent mixtures, in order to determine if this induced ordering or phase separation of the detergent [Fig. 2 ▸, blue circles; unsubtracted curves in Fig. S4(*b*)]. No additional Bragg peaks or signs of detergent micellization or aggregation were observed, indicating that even after precipitant addition, the detergents remained dispersed in the LCP bilayer. Again, slight changes to the amplitudes of the residual Bragg peak were noted due to the change in the solvent SLD from the precipitant addition and possibly also from potential changes to the degree of ordering in the LCP [Fig. S4(*c*)].

### 2.4. Measurement of SANS from the bR monomer in solution   

**Figure 3:**
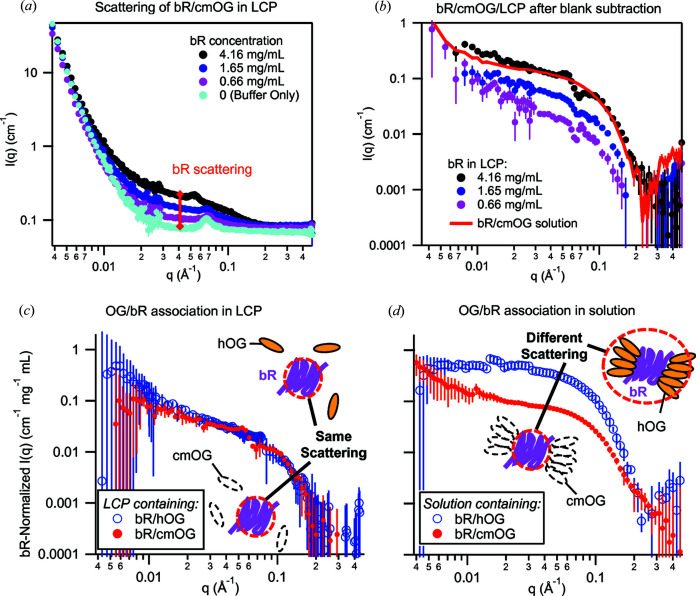
Association of bR and OG in LCP. (*a*) SANS of a concentration series of bR/cmOG in LCP, compared with blank LCP. (*b*) Blank LCP curve subtracted to obtain the scattering from bR alone, which was compared with bR solution scattering. (*c*) Subtracted curves normalized by bR concentration and averaged. Error bars are the standard deviation of the distribution of individual normalized curves prior to averaging. This procedure was performed for bR/cmOG (concentrations of 0.66, 1.7 and 4.2 mg ml^−1^) and bR/hOG (1.3, 1.4, 2.1, 2.5, 3.2 and 5.2 mg ml^−1^). Contrast-matching of OG has no observable effect on the normalized scattering, indicating that OG is not associated with bR in any significant amount in the LCP. (*d*) Buffer-subtracted and normalized scattering of bR/cmOG (0.8, 1.7, 5.2, 6.8 and 10.4 mg ml^−1^) and bR/hOG (0.9, 1.1 and 1.47 mg ml^−1^) in solution. The intensity scale is the same as for (*c*). Unlike the LCP case, in solution, contrast-matching of OG causes a pronounced change in scattering intensity, indicating association of the detergent with bR.

In addition to detergent micelles, we also studied the incorporation of bR into the LCP, starting from monomeric protein solubilized in octyl glucoside (OG). The common assumption is that bR would be incorporated into the LCP as a ‘bare’ monomer (*i.e.* without bound detergent) (Caffrey, 2000 ▸). Therefore, we first measured SANS from bare bR monomers in solution for comparison. Since monomeric bR must, however, be maintained in solution as a bR/OG complex, scattering from the bare monomer can only be obtained by contrast-matching to eliminate scattering from the detergent. In this case, unlike the case of the LCP, we attempted to perfectly match out the detergent by matching the head SLD to the tail SLD using mixtures of deuterated and non-deuterated detergents. The chemical structure of OG allows us to achieve this match at an SLD equal to that of 100% D_2_O. To do so, we prepared a mixture of 54.6% fully deuterated, 36.4% tail-deuterated and 8.9% non-deuterated OG (mole percentages). Contrast-matching was verified by performing SANS on 3%(*w*/*v*) hOG and cmOG in D_2_O; scattering from cmOG was uniformly eliminated [Fig. S3(*b*)]. After isolating monomeric bR solubilized in cmOG by SEC (Fig. S7), SANS of the bare monomer was measured in 100% D_2_O buffer [Fig. 3 ▸(*b*), solid curve]. We note that it is also possible to contrast-match hOG (which is far less expensive than cmOG) in roughly 17% D_2_O; however, due to the high neutron incoherent scattering from H_2_O [Fig. S3(*b*)], it is impractical to measure low concentrations of bR monomer in contrast-matched hOG micelles. Very high concentrations of bR in contrast-matched hOG could, in principle, be measured, but structure factor effects become significant at such concentrations [*S*(*Q*) is no longer 1], preventing the direct measurement of the bR form factor. Thus, the use of cmOG was the only route to this measurement.

### 2.5. Distribution of bR incorporated into the LCP   

After measuring scattering from bR monomers in solution, bR prepared in cmOG or hOG was mixed with dMO to form the cubic phase, and additional SANS measurements were carried out to measure the distribution and/or aggregation state of the protein and detergent. While OG was not found to form aggregated structures on its own in the LCP (see preceding section), it is conceivable that such structures could form in the presence of bR (*e.g.* associated with the bR). Therefore, it was important to compare the difference in scattering between bR/hOG and bR/cmOG in the LCP. Any observed scattering in bR/cmOG LCP samples would be related to the protein bR alone, since both the detergent cmOG and lipid dMO would be contrast-matched. Scattering in bR/hOG LCP samples, however, would be attributed to both the protein bR and any OG aggregates or micelles; since hOG was not contrast-matched, it would produce observable scattering if aggregated structures were formed, either alone (as discussed in Section 2.3) or in combination with bR.

Scattering from bR was clearly observed upon incorporation of bR/cmOG into the LCP [Fig. 3 ▸(*a*)]. As discussed in Section 2.2, the LCP matrix was not perfectly matched, leaving some small residual scattering at intermediate *q* along with significant very low *q* tails of the air bubble scattering. In order to investigate the origin of the residual Bragg peaks seen in Fig. 3 ▸(*a*), and particularly the apparent shift in the position of the peak at high bR concentration, these samples were brought away from the LCP match condition by replacement of 1/3 of the D_2_O buffer with H_2_O buffer (leaving the LCP dispersion in the cuvette), and SANS measurements were repeated (Fig. S8). By removing the solvent from the match point of the LCP/cmOG (about 100% D_2_O) nearer to the match point of the bR (about 42% D_2_O), Bragg peaks arising from lipidic structures would be enhanced, and those arising from protein structures suppressed. All Bragg peaks increased greatly in intensity upon addition of H_2_O to the sample, indicating an origin from lipidic structures, rather than from ordered protein structures. The onset of a bR concentration-induced phase change in the LCP is also apparent by the shift in the *q* position of the Bragg peak at the highest bR concentration.

In order to remove the LCP contribution to the sample scattering and observe the form factor scattering of bR, a ‘blank’ LCP sample (of identical composition but without bR) was prepared (light blue curve), and its scattering subtracted from the bR/LCP samples [Fig. 3 ▸(*b*)]. The subtracted bR/LCP curves, containing mainly the bR scattering contribution, were then compared with the solution scattering of monomeric bR (solid red curve). The curves were found to have the same shape, indicating that the distribution and aggregation state of bR in LCP were the same as for bR in solution. The scattering from bR in LCP was also compared with the theoretical scattering from monomeric and trimeric bR (Fig. S9) as calculated from the crystal structure (PDB entry 1c3w; Luecke *et al.*, 1999 ▸) using *CRYSON* (Svergun *et al.*, 1998 ▸), with calculated scattering curves placed on an absolute scale using the *SASSIE Contrast Calculator* (Sarachan *et al.*, 2013 ▸). No evidence of significant additional bR aggregation upon incorporation into the LCP (an increase in the magnitude of scattering and shifts in the SANS curve turnover to lower *Q*), non-uniform distribution [which would lead to an *S*(*Q*) ‘interaction peak’] or ordered structures (which, as discussed, would result in new Bragg peaks that would be suppressed upon addition of H_2_O) was observed. Scattering of bR in LCP was consistent with the theoretical scattering of monomeric bR containing a small proportion of aggregate (roughly 4% of the total protein) as estimated by fitting a fractal aggregate model.

Next, the scattering of bR/cmOG/LCP was compared with that of bR/hOG/LCP [Fig. 3 ▸(*c*)]. A similar blank LCP subtraction procedure (not shown) was performed for bR/hOG/LCP, and all scattering curves were normalized (*i.e.* divided by the calculated concentration of bR in the beam-illuminated volume in milligrams per millilitre). If the subtraction is valid, and the only remaining scattering is from bR, normalization should cause curves obtained from different bR concentrations to coincide (assuming structure factor effects are not too great). This was indeed found to be the case. These curves from different bR concentrations were averaged for the purposes of display in Fig. 3 ▸(*c*). Error bars are plus or minus one standard deviation of the distribution of the individual normalized concentration series curves and thus capture the uncertainties related to errors in the bR concentration used for normalization. The bR/cmOG/LCP and bR/hOG/LCP curves coincided in shape and absolute intensity, indicating that OG was not contributing to the scattering object. This implies that the OG was not significantly present in aggregated states, either alone or in complex with bR.

Finally, the scattering of bR/cmOG and bR/hOG in solution (as protein–detergent complex) was compared [Fig. 3 ▸(*d*)]. An analogous subtraction and normalization procedure was used. Instead of subtracting the blank LCP, a buffer blank with the same concentration of free OG micelles was subtracted, so that scattering from the bR/OG complex could be obtained without a contribution from free micelles (this is not strictly necessary in the cmOG case since free micelles are matched and do not scatter; only the incoherent background subtraction needs to be subtracted). Unlike the LCP case, the shape and normalized absolute intensity of the bR/hOG scattering is strikingly different from the bR/cmOG scattering (shifted to lower *q*, and significantly stronger scattering). Comparison of Figs. 3 ▸(*c*) and 3(*d*) shows, unambiguously, that OG is associated with bR in solution, but does not remain associated (with bR or with itself) upon incorporation into the LCP.

### 2.6. Crystallization screening of bR in different detergents   

**Figure 4:**
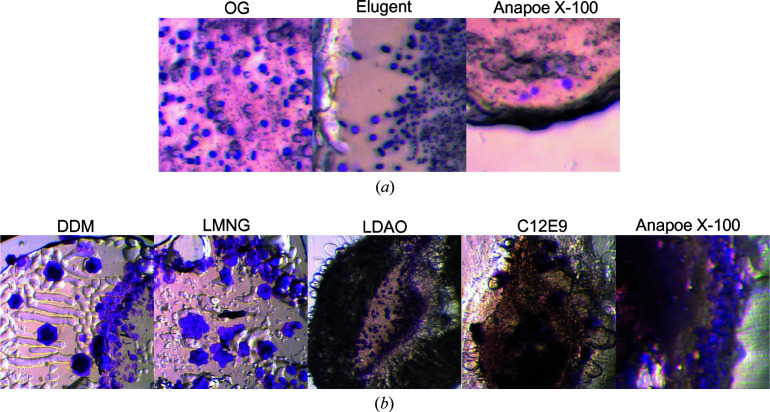
LCP crystallization screening of bR in different detergents. (*a*) Detergents that allowed direct solubilization and LCP crystallization of bR. (*b*) Detergents not suitable for bR solubilization, but which nevertheless allowed crystal formation when spiked into the LCP. In this case bR was first solubilized in OG and mixed into the LCP before adding a fivefold mass excess (relative to OG) of the additional detergent.

We attempted crystallization trials of bR in different detergents in order to relate scattering to crystallization behavior. Not all detergents were found to be suitable for the extraction and maintenance of stable bR in solution, with DDM, OGNG, LMNG and C12E9 giving low extraction yields, whereas zwitterionic detergents such as LDAO and FC10 caused denaturation (observed as a color change upon dissociation of the retinal cofactor). However, crystals could be obtained in all detergents that can extract and maintain bR in stable solution [Fig. 4 ▸(*a*)]: octyl glucoside, Elugent and Triton X-100. We also verified that the use of D_2_O/dMO as opposed to the usual H_2_O/hMO compounds did not appear to affect crystallization; crystals of similar size and morphology were obtained at the same precipitant concentrations regardless of deuteration.

For the other detergents, it is possible that crystallization failed, not because of a fundamental incompatibility of the detergent with LCP crystallization, but because of the lack of suitability of the detergent prior to incorporation of the protein into LCP. Therefore, to investigate detergent suitability in the LCP independent of the protein solution behavior, we also performed crystallization studies of OG-solubilized bR incorporated into LCP that was then spiked afterwards with a fivefold mass excess of other detergents. This resulted in several additional detergents found to allow crystallization [Fig. 4 ▸(*b*)].

## 3. Discussion   

The crystallization of membrane proteins from LCP is a technique that has been empirically valuable for producing crystals, but many assumptions about the mechanisms for this process have not been subjected to extensive experimental verification. The most common hypothesis (Caffrey, 2000 ▸) is that the membrane protein and detergent ‘dissolve’ into the LCP and then diffuse freely, allowing membrane proteins to form crystals upon precipitant addition. The formation of crystals requires diffusion, and this has been observed directly in fluorescence recovery after photobleaching (FRAP) experiments (Cherezov *et al.*, 2008 ▸). However, the existence of a diffusible protein species does not preclude the possibility of transient ‘preferred sites’ or aggregated structures in equilibrium being formed upon incorporation of proteins into the LCP. In fact, FRAP studies have sometimes suggested the presence of a non-mobile protein species, or of subpopulations with different mobilities (Cherezov *et al.*, 2008 ▸). Interpretation of diffusion rates is also not straightforward in the crowded LCP environment, and the structural nature (*e.g.* monomers, trimers, aggregates) of populations of differing mobility is therefore not directly known.

An additional line of evidence suggesting the possibility of intermediate structures on the pathway to crystallization was the observation of lamellar extensions near the edges of growing protein crystals (Cherezov & Caffrey, 2007 ▸). However, it is not clear if such structures are present (perhaps transiently) in the absence of crystals: for instance, when protein is initially incorporated into the LCP before precipitant addition, or in the early stages of crystal nucleation. Instead, since LCP protein crystals have (thus far) always been observed to have lamellar (‘type I’) packing (Caffrey, 2015 ▸), these lamellar extensions may simply be the natural result of connecting the edge of an LCP protein crystal with the rest of the bulk cubic phase.

Furthermore, none of these studies address the distribution in LCP of the detergent – the other key component in almost all membrane protein crystallization studies. Detergents could, in principle, maintain some association with the membrane protein or form other types of phase-separated structures in LCP, especially upon precipitant addition. As a practical matter, if detergents were to remain associated with the membrane protein, or to form phase-separated structures, then one would expect them to be influential in the crystallization process. Conversely, if detergents generally dissolve into freely diffusing monomers in the LCP bilayer, then one would expect their identity to be less important to crystallization (although their effect on the bulk properties of the cubic phase, such as its lattice parameter, might still matter). Generally, in the LCP, detergents are present in small proportions compared with the lipid itself. We therefore hypothesized that, in the absence of protein associations or the formation of significant phase-separated structures, detergent identity would be expected to have a minor effect on LCP crystallization of proteins. This idea could potentially offer great savings in both time and expense for crystallization studies; rather than needing to optimize a detergent for both solution stability and crystallization, only the former would need to be considered. Furthermore, inexpensive and heterogeneous detergent mixtures could be considered for use such as Triton X-100 and Elugent as in this study. To the best of our knowledge, such detergents are not commonly employed in LCP crystallization experiments, possibly due to habits acquired from *in surfo* crystallization practices.

In our SANS studies, we found that indeed many of the common assumptions were validated. All of the detergents we tested dissociated when initially incorporated into the LCP and were also not seen to form phase-separated structures upon addition of a high salt precipitant. The main observable effect of detergent identity is on the LCP lattice parameter, with some detergents causing more swelling of the LCP than others. One could indeed imagine this impacting crystallization, for example by causing the water channels to be large enough to allow diffusion of a larger membrane protein. However, the lattice parameter can also be controlled within a wide range by simply varying the amount of detergent in the final sample, rather than the detergent identity itself. For instance, in a high-salt crystallization condition, it has been found (Misquitta & Caffrey, 2003 ▸) that the OG concentration can set the lattice parameter within a range of 91–151 Å, and LDAO within 85–136 Å (at the upper limit, transition from 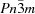 to 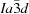 or a mixed phase occurs). Finally, in the particular model system of bR/OG in monoolein LCP without precipitant, the membrane protein remained as isolated monomers which were not observed to localize in any set of periodic ‘preferred sites’ in the LCP (which would cause intensity modulation of the LCP Bragg peaks), or to form other periodic structures like 2D crystalline patches (which would lead to new Bragg peaks in distinct positions), nor did it remain associated with detergent (which would cause a shift in *Q* and in the intensity of the bR scattering curve). The effect of precipitant on bR in LCP is beyond the scope of the current work and will be addressed in a future publication.

Finally, and consistent with the dissociation of most detergents in the LCP, we found experimentally that bR crystals could be grown from several detergents with widely different properties (*e.g.* CMC, micelle size/shape). To the best of our knowledge, this is the first reported crystallization of bR in LCP using inexpensive mixtures like Triton X-100 or Elugent. Our results suggest that such detergents should be tried for new membrane protein targets where LCP crystallization is to be attempted. Furthermore, in screening detergents for these membrane proteins, the aim should be to find detergents that support the best possible extraction efficiency and protein stability during purification, without regard to the size of the micelle or its suitability for *in surfo* crystallization.

## 4. Materials and methods   

### 4.1. Solutions and reagents   

Detergents were obtained from Anatrace^1^ unless otherwise specified. Non-deuterated monoolein (hMO) was obtained from Nu Chek Prep. Product numbers and detergent abbreviations used in this study are specified in Table S3, with estimated molecular volumes and SLDs provided in Table S2, and chemical structures shown in Fig. S10. Other buffers and reagents were obtained from Sigma unless otherwise specified. Highly deuterated monoolein (dMO) was synthesized as follows: firstly, highly deuterated oleic acid was prepared as described (Darwish *et al.*, 2013 ▸). Glycerol-d_8_ (98% deuterated) was obtained from Sigma. According to a published method for the unlabeled analog (Srisiri *et al.*, 1998 ▸), an isopropylidene acetal protecting group was introduced to glycerol-d_8_ in order to facilitate *sn*-1 esterification, which was subsequently achieved under Steglich conditions. After the esterification reaction, the protecting group was removed to provide highly deuterated 1-monoolein (dMO). Some acyl migration was observed, resulting in the presence of a small amount of highly deuterated 2-monoolein (a resonance at 3.81 p.p.m. in the ^2^H NMR spectrum is assigned to the two glycerol methylene moieties in the symmetrical molecule). The ready occurrence of acyl migration observed is in accordance with that reported for the unlabeled compound (Mattson & Volpenhein, 1962 ▸; Dawson *et al.*, 1989 ▸). Overall deuteration of dMO was found to be 93.2 ± 2% by mass spectrometry. With the assumption that the glycerol head group remained at 98% deuteration after dMO synthesis, this implies that the tail deuteration was 92.5%. The Certificate of Analysis of the dMO is provided in the supporting information.^1^

Solubilization buffer for bR consisted of 25 mmol l^−1^ NaH_2_PO_4_ titrated to pH 6.9 with KOH and 40 mmol l^−1^ OG (1.2% *w*/*v* for hOG). SEC buffer was prepared similarly, but instead titrated to pH 5.6 with 1.35 mmol l^−1^ KOH. For contrast-matching of dMO, SEC buffer was prepared using D_2_O instead of H_2_O, and the same concentration of buffer salts (25 mmol l^−1^ NaH_2_PO_4_, 1.35 mmol l^−1^ KOH) without additional pH adjustment. Solutions using contrast-matched OG mixture (cmOG) consisted of 3.3 mmol l^−1^ non-deuterated OG, 14.6 mmol l^−1^ tail-deuterated OG and 22.1 mmol l^−1^ fully deuterated OG. Low-salt buffer consisted of SEC buffer without detergent. Precipitant buffer was prepared by mixing NaH_2_PO_4_ (or NaD_2_PO_4_) with K_2_HPO_4_ (or K_2_DPO_4_) at a fixed molar ratio of 73.2:26.8 without additional pH adjustment, which gives a pH of about 5.6 at total phosphate concentrations of 2.0 mol l^−1^ in H_2_O. NaD_2_PO_4_ and K_2_DPO_4_ stock solutions were prepared from the ^1^H compounds by hydrogen exchange, using repeated dissolution in D_2_O and lyophilization. Precipitant concentrations are always specified with respect to total phosphate, with the ratio of sodium and potassium salts fixed as specified above. Buffer and precipitant solutions were always prepared with fixed ratios of buffer salts, as specified above, without attempting to correct for the effects of ionic strength or D_2_O fraction on the pH/pD. Deuterated solutions were used for SANS (Fig. 2 ▸ and 3 ▸) whereas crystallization screening was carried out using non-deuterated solutions (Fig. 4 ▸).

### 4.2. Expression and purification of monomeric bR   

bR was expressed in *Halobacterium salinarum*, followed by isolation of the bR-containing ‘purple membrane,’ solubilization with OG and purification by SEC following established protocols (Cleveland & Kelman, 2015 ▸; Dencher & Heyn, 1982 ▸). Briefly, *H. salinarum* was grown in 10 l batches in illuminated shake flasks at 310 K for 5 d before centrifugal harvesting of the cells. Cell pellets were resuspended in water (containing protease inhibitors and DNase) using a Dounce homogenizer, a process which causes cell lysis. Membranes were then washed using 2 to 3 cycles of ultracentrifugal pelleting and resuspension in water. Finally, the purple membrane was isolated by sucrose gradient ultracentrifugation and stored as a suspension at 277 K for up to 6 months. Stored purple membrane suspensions contained bR at concentrations of 2–8 mg ml^−1^ as determined by optical absorbance at 568 nm (using an extinction coefficient of 62700 l mol^−1^ cm^−1^) (Rehorek & Heyn, 1979 ▸). Detailed protocols for growth, harvest and membrane isolation were essentially as described (for unlabeled bR) (Cleveland & Kelman, 2015 ▸).

To purify monomeric bR by SEC, the purple membrane suspension was adjusted to the composition of solubilization buffer by adding water, buffer and detergent, using a final volume such that the total mass of OG in solution was 20 times the total mass of bR. Solubilization was performed for 12 to 24 h at room temperature in the dark, at which point insoluble material was removed by ultracentrifugation. bR was then concentrated to about 20 mg ml^−1^ using 50 kDa Amicon spin concentrators (Millipore). For SEC purification, up to 0.5 ml of concentrated bR solution (10 mg of bR) was injected onto a Superdex 200 Increase column (GE Healthcare) equilibrated in SEC buffer. The column was run at a flow rate of 0.3 ml min^−1^ at 277 K in the dark. Multiple injections were used as necessary to process amounts of bR solution above 0.5 ml. Absorbance was followed at 550 nm (and 620 nm to avoid detector saturation) and monomeric bR-containing fractions were pooled and spin-concentrated to the desired final concentration (see Fig. S7 for a typical SEC trace).

### 4.3. Incorporation of detergents and bR into the LCP for SANS   

Detergent-only solutions were prepared at 3%(*w*/*v*) in D_2_O for solution scattering and for incorporation into the LCP (Figs. S1 and 2 ▸). Solutions were prepared by adding 0.97 ml of D_2_O per 0.03 g of detergent (*i.e.* correction for the partial specific volume of the detergent was neglected). Detergents supplied as H_2_O solutions (*e.g.* Anapoe C12E9) were lyophilized to give the appropriate mass of detergent before adding D_2_O. To prepare the LCP, a solution containing detergent and/or bR (at up to 20 mg ml^−1^) was initially mixed at a volume fraction of 0.4 to 0.5 with dMO that had been melted at 315 K. A two-syringe mixer was used as described (Caffrey & Cherezov, 2009 ▸). An additional volume of low-salt buffer in D_2_O was then introduced using one of the syringes and remixed, such that the mixture contained a buffer volume fraction of 0.75. This results in excess hydration of the LCP, and thus gives a two-phase suspension of fully hydrated LCP in low-salt D_2_O buffer (see Fig. S11). For SANS, a needle was attached to the syringe containing the mixture, which was then injected into the center of a cuvette that had been pre-filled with low-salt D_2_O buffer. Since cuvettes were not entirely filled with the mixture, care was taken to ensure that it was placed entirely in the center of the cuvette, so that the entire volume of the mixture was contained within the beam (the volume near the edges of the cuvette is not illuminated by the beam). The LCP is sticky enough to remain in place after injection into a cuvette. In some cases with LCP outside the cuvette center, the fraction of LCP in the beam was measured by optical imaging and densitometry, and used to normalize the scattering intensities by the amount of LCP in the beam.

It is important to understand that the LCP matrix is paste-like and remains so upon incorporation of the protein and/or detergent. Thus, creating a homogeneous mixture of the protein or detergent solutions and LCP requires vigorous mixing, with high shear rates, achieved via the syringe-mixing mentioned above. Vigorous mixing will in general introduce air and cavitation events which will eventually produce a large amount of micrometre-sized bubbles. As shown in Fig. S5, these would be nearly invisible in H_2_O (with an SLD near 0, like air) but cause strong, power law, low-*Q* scattering in D_2_O. This is rarely an issue in typical liquid samples, since bubbles will float out of solution. They can stick to glass surfaces of sample cells, but the comparatively small amount is only an issue with very weak scatterers, and furthermore can be removed by centrifugation or by careful clearing procedures immediately prior to sample loading to remove nucleation sites if necessary. For viscous materials, degassing or centrifugation (or in many cases, time) will generally remove the bubbles. Degassing at elevated temperature where the sample is not as viscous can also be effective. If the samples do not require vigorous agitation/mixing, the number of bubbles is likely to be small and only a problem for weak scatterers. In these cases degassing the components will often reduce or eliminate the problem. Sonication can also be used, but only when it is not likely to alter the sample itself, which is not often the case and thus rarely useful. Unfortunately, the paste-like nature of the samples here, before and after vigorous mixing, renders these techniques ineffective, and the high temperatures required to attempt to fluidize the LCP did not seem reasonable after incorporation of the protein.

### 4.4. Small-angle neutron scattering, data collection and analysis   

Neutrons do not interact with the electron cloud the way X-rays do, but with the nucleus. Hence, while X-ray scattering power increases with increasing *Z* (electrons in the atom), neutron scattering power varies in a seemingly random way across the periodic table and, most notably, can vary dramatically from isotope to isotope within a single element (Sears, 1992 ▸). Of particular note is the vast difference between hydrogen and deuterium scattering power which makes neutrons particularly useful for the study of hydrogenated materials. One drawback is the existence of the incoherent scattering component, which provides no structural information, but is a flat *q*-independent background scattering. Again, the fraction of coherent versus  incoherent is element- and isotope-dependent, with hydrogen being one of the largest incoherent scatterers. This leads to the incoherent background from H_2_O being an order of magnitude higher than that of D_2_O. For this reason, we chose to deuterate the majority component of our samples (LCP and/or buffer), with the minority component (*e.g.* protein) hydrogenated, for maximum contrast with minimum background. An interesting consequence of this is that air pockets, whose scattering power is close to that of water, will only be problematic in precisely the deuterated matrices of choice for most scattering experiments, as discussed in Section 4.3 and Fig. S5. Finally, since SANS only probes larger structures (down to about 1 nm), the atomic scattering power (given in terms of atomic scattering length) can be replaced by an average scattering length density (SLD), in which all the atomic scattering lengths in a volume of interest are summed and divided by that volume to normalize. The SLD is directly related to the neutron index of refraction. It is thus only changes in coherent SLD that lead to coherent scattering, which contains structural information of interest.

SANS data were collected on beamlines NG7, NGB and NGB30 (Glinka *et al.*, 1998 ▸) at the NIST Center for Neutron Research (NCNR), Gaithersburg, MD. Instrumental parameters are specified in the headers of the reduced datasets, which have been deposited in the SASBDB. Samples were inserted into cylindrical Hellma quartz Suprasil cuvettes with 1 mm path length, and data were collected at 295 K. SANS data were reduced and placed on an absolute scale using software developed by the NCNR (Kline, 2006 ▸). Error bars on SANS plots are plus or minus one standard deviation and represent the uncertainty due to counting statistics.

Micelle scattering data were analyzed using the software *SasView* (Doucet *et al.*, 2017 ▸) with the built-in solid ellipsoid model used to fit the scattering data of all detergents. This was done for simplicity and uniformity in quantifying micelle size/shape, even though in some cases, other models may have been more appropriate (*e.g.* the very long micelle of AX-114 at 295 K is better fit by a flexible cylinder model). Fits were performed using the *DREAM* optimizer in *SasView* (Fig. S2), which provides an estimate of parameter correlations and uncertainties (Vrugt *et al.*, 2009 ▸). Structure factor effects were included using a ‘hard sphere’ model, with the effective hard sphere interaction radius constrained during fitting to the average radius of curvature of the ellipsoid. In most cases, the volume fraction and SLD of the micelle were fit independently. These parameters are strongly correlated, but independent fits are possible due to the inclusion of structure factor effects, which affect the shape of the scattering curve at low *q* as a function of volume fraction. For ‘long’ micelles, the hard sphere structure factor model was not suitable and was omitted, with volume fractions simply fixed at 0.03. Although the volume fraction of detergent is known *a priori* to be roughly 0.03 based on preparation of 3% *w*/*v* solutions, the micelle volume fraction can be different due to the inclusion of significant amounts of water into the micelle. Compared with an analysis where the volume fraction is fixed at 0.03, this has the effect of raising the micelle SLD and thus reducing the contrast (detergents have calculated SLDs in the range 0–2 × 10^−6^ Å^−2^, with admixed D_2_O having an SLD of 6.4 × 10^−6^ Å^−2^), increasing the micelle dimensions and hence the total volume fraction of micelle, as the micellar volume now includes some volume of additional water. The calculated model intensities were smeared according to the *Q*-resolution of the data using built-in procedures in *SasView*.

### 4.5. Screening different detergents for LCP crystallization of bR   

Two types of screening experiments were performed on a selection of detergents (OG, DDM, Elugent, Anapoe X-100, C12E9, LMNG and LDAO). Initially, bR was solubilized and incorporated into the LCP using each detergent of interest before attempting crystallization. Solubilization of bR was performed in exactly the same way as for OG (see above), but omitting the SEC purification steps and proceeding directly to the concentration and LCP incorporation steps. Crystallization screening was performed by placing 0.2 l droplets of bR/LCP, overlaid with 1 l of precipitant solution (1 mol l^−6^ to 3 mol l^−1^ Na/K Phosphate pH 5.6) between glass coverslips separated by nine-well adhesive spacers as described (Caffrey & Cherezov, 2009 ▸). Crystal growth usually occurred over 1–7 d.

In the second type of screening (*i.e.* ‘spike-in’ screening), bR was first extracted in OG, concentrated and SEC-purified as usual in order to remove any excess OG. The protein was then concentrated to about 30 mg ml^−1^, incorporated into LCP by mixing 1:1 with monoolein, and 0.2 l droplets dispensed onto coverslips as usual. The amount of OG carried over with the bR was about 6.7 g per droplet, as determined by thin-layer chromatography. At this point, an additional 0.34 l of 100 mg ml^−1^ detergent solution, containing the detergent of interest, was dispensed onto the bR/LCP droplets (thus adding a fivefold mass excess, 34 g, of the spike-in detergent relative to the OG). Droplets were watched until excess liquid had just disappeared and only a single body of LCP remained (less than 5 min). Finally, 1 l precipitant solutions were overlaid on the droplets, which were sandwiched between a second glass coverslip as usual and observed for crystal growth.

## 6. Data availability   

Data have been deposited in the Small-Angle Scattering Biological Data Bank (SASBDB) under accession codes SASDJD4 (solution datasets) and SASDJE4 (LCP datasets). A single ‘index’ dataset of bR in solution or LCP, respectively, has been deposited under each accession code, with all additional datasets available as an additional downloadable archive. 

## Supplementary Material

Supplementary figures. DOI: 10.1107/S2052252520013974/fs5185sup1.pdf

Certificate of Analysis for dMO. DOI: 10.1107/S2052252520013974/fs5185sup2.pdf

## Footnotes

1 Certain trade names and company products are identified in order to specify adequately the experimental procedure. In no case does such identification imply recommendation or endorsement by the National Institute of Standards and Technology, nor does it imply that the products are necessarily the best for the purpose.
