# Supplementary material for: Direct localization of detergents and bacteriorhodopsin in the lipidic cubic phase by small-angle neutron scattering
Source: IUCrJ. 2021 Jan 1;8(Pt 1):22–32. doi: 10.1107/S2052252520013974 (PMC7792994; doi:10.1107/S2052252520013974)
Supplement: Supplementary file 1 [file m-08-00022-sup1.pdf]

IUCrJ

Volume 8 (2021)

Supporting information for article:

Direct localization of detergents and bacteriorhodopsin in the lipidic cubic phase by small-angle neutron scattering

Thomas Cleveland IV, Emily Blick, Susan Krueger, Anna Leung, Tamim Darwish and Paul Butler

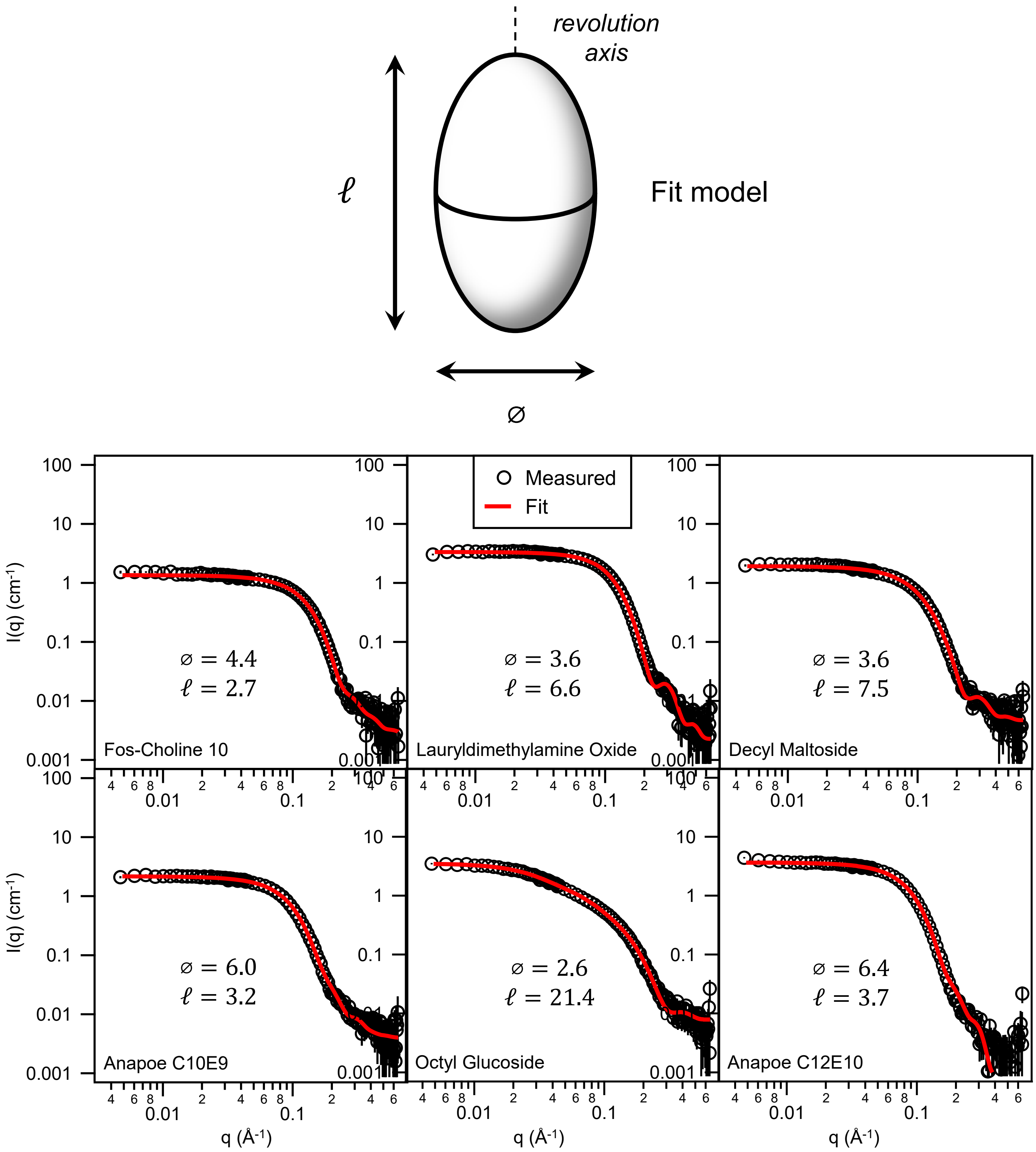

Figure S1 – Detergent micelle dimensions. SANS data were collected for detergent solutions. The data were modeled as solid ellipsoids of revolution (top) with dimensions determined by fitting. Plots are in order of calculated micelle volume from smallest to largest. Dimensions are specified in nm. See Table S1 for additional fit parameters and experimental details. Error bars are plus or minus one standard deviation and represent the uncertainty due to counting statistics.

(Figure continued next page.)

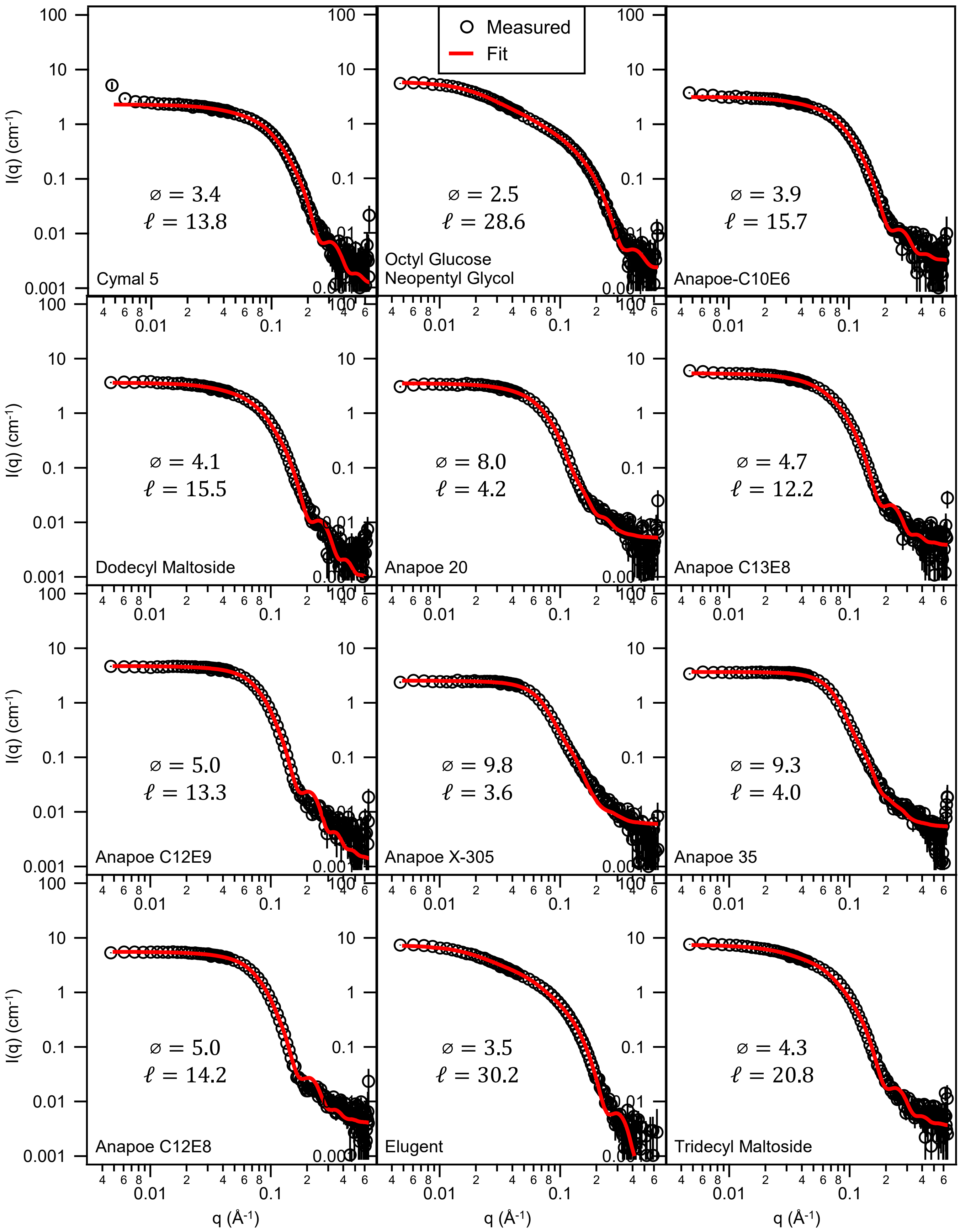

(Figure S1 – Continued from previous page.)

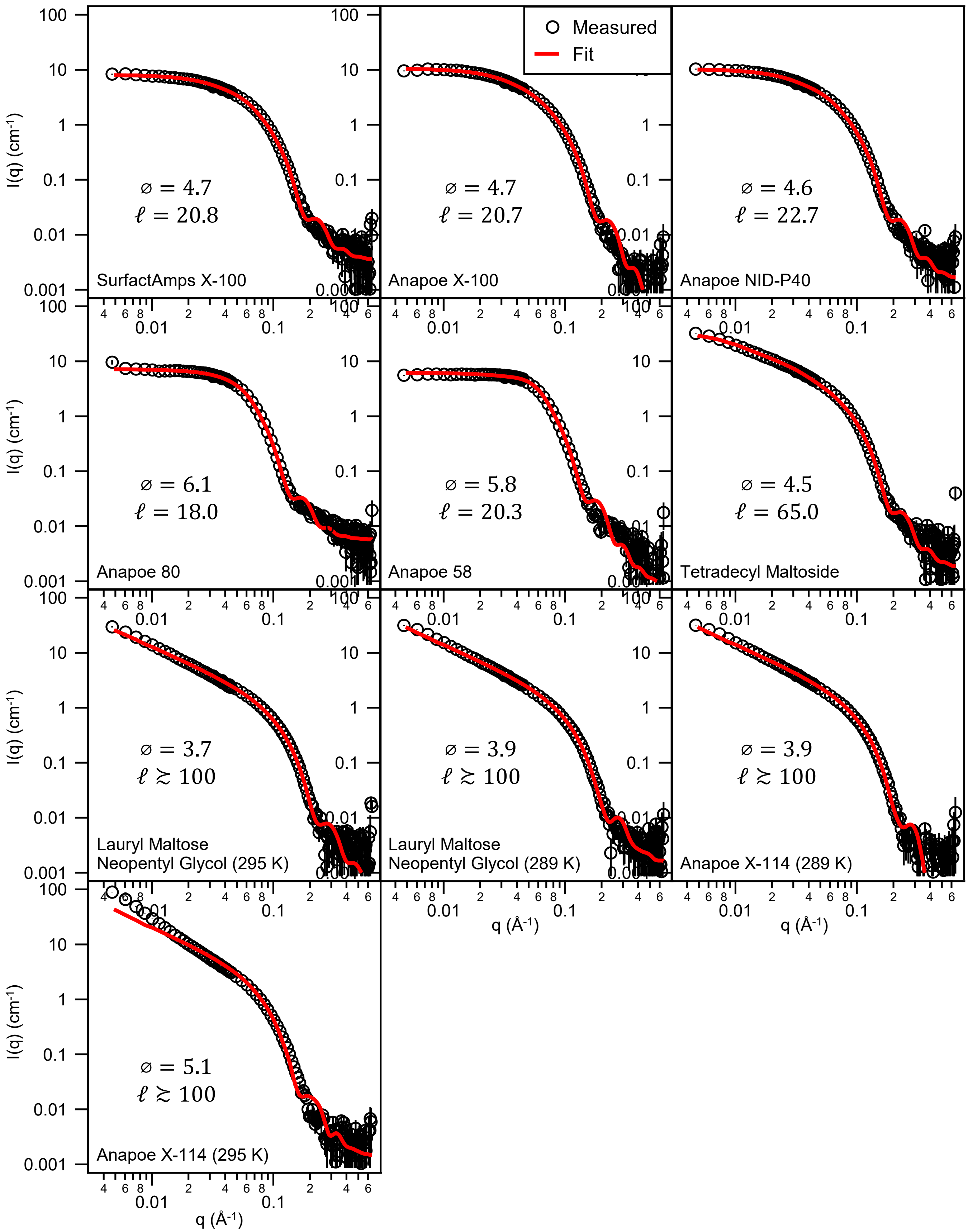

(Figure S1 – Continued from previous page.)

Detergent	Diameter (nm)	Length (nm)	Len. / Diam.	Volume (nm^3)	SLD ($\times 10^{-6}$ Å^{-2})	Vol. Frac.	Fit χ^2
FC10	4.42 ± 0.02	2.72 ± 0.02	0.61 ± 0.01	27.8 ± 0.4	1.6 ± 0.1	0.027 ± 0.001	3.9
LDAO	3.633 ± 0.007	6.55 ± 0.07	1.8 ± 0.02	45.3 ± 0.5	1.76 ± 0.07	0.052 ± 0.002	4.5
DM	3.58 ± 0.01	7.55 ± 0.05	2.11 ± 0.01	50.6 ± 0.4	2.37 ± 0.01	0.03 (fixed)	5.1
C10E9	6.03 ± 0.03	3.2 ± 0.02	0.53 ± 0.004	60.9 ± 0.7	2.3 ± 0.1	0.027 ± 0.001	2.3
OG	2.643 ± 0.008	21.4 ± 0.3	8.08 ± 0.1	78 ± 1	1.5 ± 0.1	0.022 ± 0.001	3.1
C12E10	6.42 ± 0.03	3.71 ± 0.02	0.578 ± 0.004	80 ± 0.8	1.6 ± 0.1	0.025 ± 0.001	4.6
Cymal 5	3.351 ± 0.008	13.8 ± 0.2	4.13 ± 0.06	81 ± 1	3.78 ± 0.02	0.076 ± 0.001	4.7
OGNG	2.548 ± 0.006	28.6 ± 0.2	11.21 ± 0.09	97.1 ± 0.8	1.88 ± 0.01	0.03 (fixed)	2.3
C10E6	3.937 ± 0.008	15.7 ± 0.2	3.99 ± 0.06	127 ± 2	3.97 ± 0.02	0.08 ± 0.002	3.3
DDM	4.115 ± 0.008	15.5 ± 0.2	3.77 ± 0.06	138 ± 2	3.85 ± 0.03	0.073 ± 0.002	4.8
A20	7.99 ± 0.05	4.24 ± 0.04	0.53 ± 0.01	142 ± 2	2.2 ± 0.1	0.016 ± 0.002	3.3
C13E8	4.72 ± 0.01	12.2 ± 0.2	2.58 ± 0.04	142 ± 2	2.7 ± 0.1	0.038 ± 0.002	4.5
C12E9	5.02 ± 0.01	13.3 ± 0.3	2.66 ± 0.06	176 ± 4	3.94 ± 0.03	0.094 ± 0.003	5.0
AX-305	9.79 ± 0.08	3.57 ± 0.02	0.364 ± 0.004	179 ± 3	4.58 ± 0.02	0.087 ± 0.002	4.8
A35	9.3 ± 0.2	4.03 ± 0.06	0.43 ± 0.01	180 ± 10	4.2 ± 0.1	0.09 ± 0.008	3.4
C12E8	4.992 ± 0.009	14.2 ± 0.3	2.85 ± 0.06	186 ± 4	3.78 ± 0.03	0.089 ± 0.002	4.2
Elugent	3.494 ± 0.007	30.2 ± 0.4	8.7 ± 0.1	193 ± 2	2.26 ± 0.07	0.028 ± 0.001	5.3
C13M	4.307 ± 0.006	20.8 ± 0.2	4.83 ± 0.05	202 ± 2	3.24 ± 0.03	0.059 ± 0.001	9.2
SX-100	4.65 ± 0.04	20.8 ± 1.8	4.5 ± 0.4	240 ± 20	3.3 ± 0.1	0.054 ± 0.007	3.5
AX-100	4.661 ± 0.007	20.7 ± 0.3	4.44 ± 0.06	236 ± 3	2.32 ± 0.09	0.036 ± 0.002	4.0
NP-40	4.631 ± 0.006	22.7 ± 0.3	4.9 ± 0.06	255 ± 3	2.82 ± 0.05	0.045 ± 0.001	3.7
A80	6.15 ± 0.01	18 ± 0.4	2.92 ± 0.07	355 ± 8	4.21 ± 0.04	0.08 ± 0.003	3.7
A58	5.83 ± 0.01	20.3 ± 0.3	3.47 ± 0.05	361 ± 6	4.47 ± 0.01	0.128 ± 0.001	8.0
C14M	4.509 ± 0.005	65 ± 0.8	14.4 ± 0.2	692 ± 8	1.47 ± 0.07	0.025 ± 0.001	9.5
LMNG (295 K)	3.725 ± 0.006	>100	long	long	2.42 ± 0.01	0.03 (fixed)	8.7
LMNG (289 K)	3.861 ± 0.006	>100	long	long	2.39 ± 0.01	0.03 (fixed)	10.6
AX-114 (289 K)	3.86 ± 0.006	>100	long	long	2.385 ± 0.005	0.03 (fixed)	11.0
AX-114 (295 K)	5.133 ± 0.008	>100	long	long	2.757 ± 0.005	0.03 (fixed)	119.3

Table S1 – Solid ellipsoid fit parameters. Solutions of approximately 3% (w/v) were prepared by adding 0.97 mL of D_2O to 0.03 g of detergent (i.e. correction for detergent density and volume change due to mixing was neglected). Detergents supplied as H_2O solutions (e.g. Anapoe C12E9) were lyophilized to give the appropriate mass of detergent before adding D_2O. Detergent abbreviations are defined in Table S3. The maximum dimension measurable by SANS (under the present instrument configuration) was about 100 nm. In most cases, especially when the diameter and length are within roughly 2–3 fold, both prolate and oblate solutions usually exist with very similar fit quality. In these cases, the solution with the best χ^2 is shown above, but it should be understood that small changes in data or fitting procedures can easily result in selection of the alternative solution. Total ellipsoid volume, however, is generally comparable whether the prolate or oblate solution is selected. Modeling of structure factor effects was omitted in certain cases, as discussed in Fig. S2, in which case the volume fraction was fixed at 0.03. Parameter uncertainties are 68% confidence intervals.

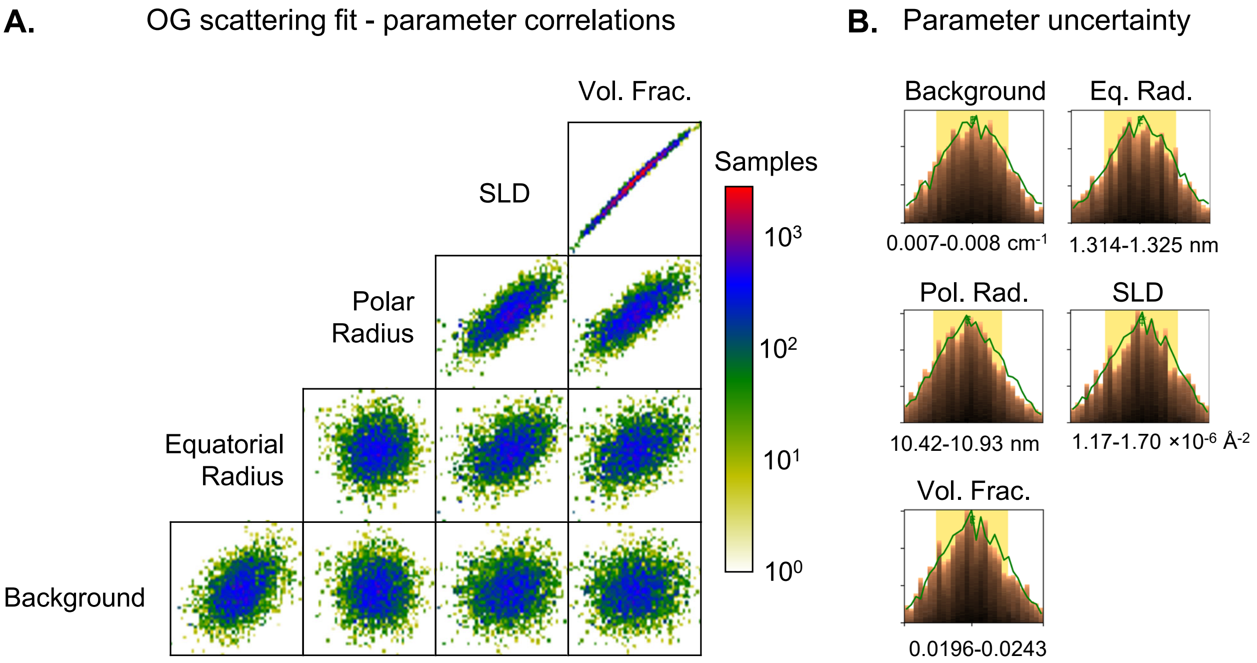

Fig. S2 – Fitting procedures for detergent micelle scattering. A solid, monodisperse ellipsoid model was used to fit the scattering data of all detergents. This was done for simplicity and uniformity in quantifying micelle size/shape, even though in some cases, other models may have been more appropriate (e.g., the very long micelle of AX-114 at 295 K is better fit by a flexible cylinder model, which better captures the low-q power law; $\chi^2 = 7$ for a flexible cylinder, as opposed to 119 for the solid ellipsoid; data not shown). Fits were performed using SasView with the DREAM optimizer (Vrugt *et al*, 2009), which provides an estimate of **(A)** parameter correlations and **(B)** uncertainties (OG is shown above as an example). The axis limits of (A) and (B) are the 95% confidence intervals, which are listed in (B). Structure factor effects were included using a "hard sphere" model, with the effective hard sphere interaction radius constrained during fitting to the average radius of curvature of the ellipsoid. In most cases, the volume fraction and SLD of the micelle were fit independently. For "long" micelles (see Table S1), the hard sphere structure factor model was not suitable and was omitted, with volume fractions simply fixed at 0.03. This was also done in certain cases where the apparent structure factor effects were judged to be too small to reasonably constrain the fit (as determined by the SLD vs. volume fraction correlation plot). The maximum dimension measurable by SANS (under the present instrument configuration) was about 100 nm. In most cases where the length and diameter are similar (within 2-3 fold of each other), both prolate and oblate solutions can be found. In this case, the solution with the best χ^2 was reported in Table S1. However, it should be understood that these solutions can be nearly indistinguishable, such that small errors or changes in fitting procedures can easily shift the choice from one to the other. Given the model simplifications, the choice of prolate or oblate solution should not be taken as certain, at least when the length and diameter are similar.

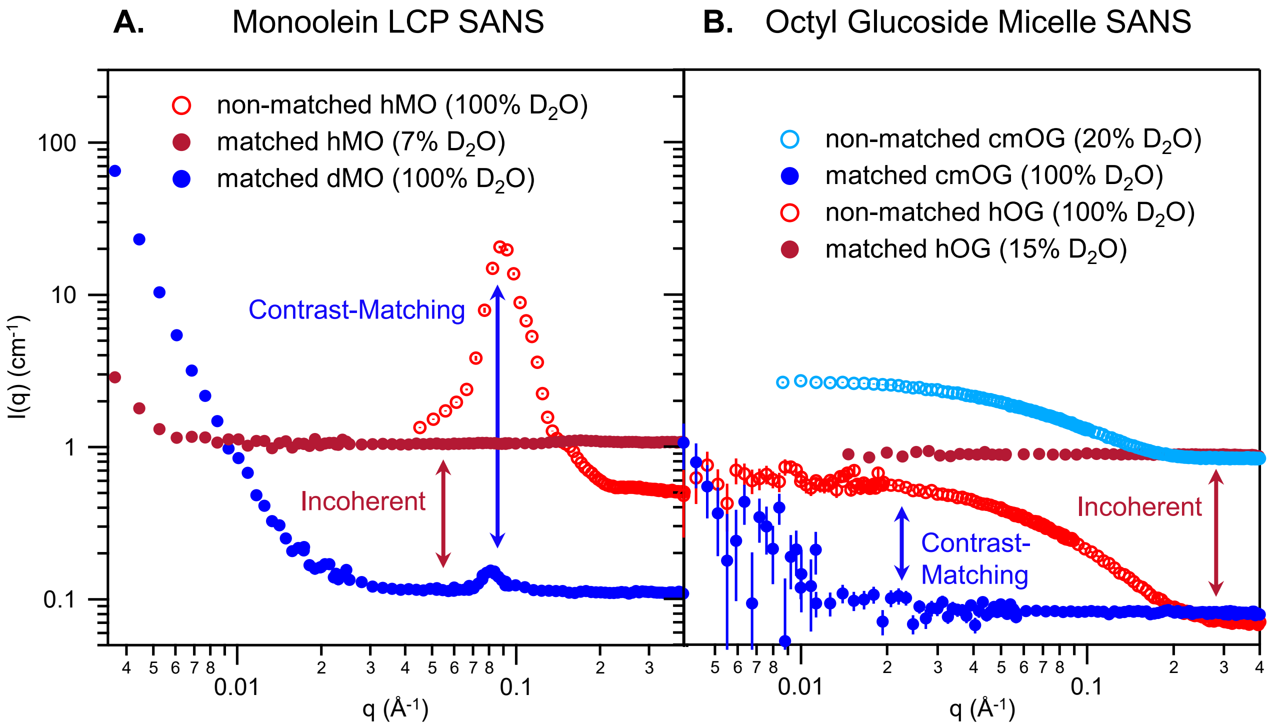

Figure S3 – Contrast matching of (A) LCP and (B) OG micelles. In 100% D_2O, scattering from the LCP is reduced by two orders of magnitude, making it possible to subtract the remaining LCP scattering when bR is included. LCP scattering is not completely eliminated since the lipid head and tail groups are not perfectly matched to each other. Scattering from cmOG micelles is, however, entirely absent at the match point of 100% D_2O (the small amount of scattering at very low-q is typical of, e.g., cuvette imperfections, and is mainly visible due to the otherwise very low amount of scattering from cmOG). It is also possible to contrast-match the non-deuterated compounds ("hMO" and "hOG") using mixtures near 100% ^1H_2O, but this leads to high incoherent backgrounds from ^1H that mask the signal of interest from bR. Error bars are plus or minus one standard deviation and represent the uncertainty due to counting statistics.

A. Without precipitant addition

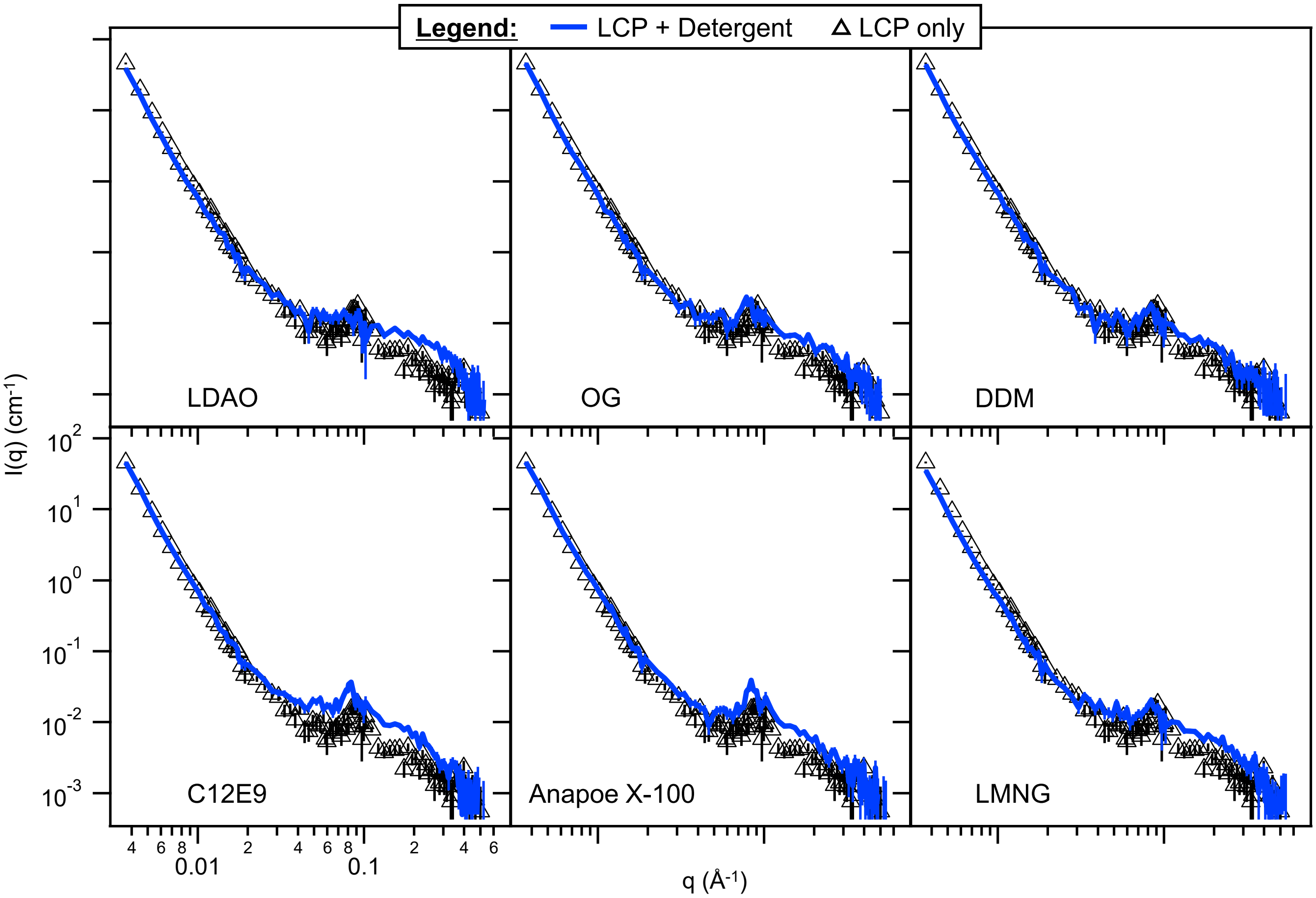

Fig. S4 – SANS curves for LCP with and without incorporated detergent. Scattering intensities on an absolute scale, without normalization for detergent concentration, are shown after subtraction of the incoherent background. Error bars are plus or minus one standard deviation and represent the uncertainty due to counting statistics. **(A)** Subtraction of the LCP-only curve (black triangles) from the LCP + detergent curves (solid blue lines), and division by the detergent concentration in mg/mL, yields the blue curves in Fig. 2. Because the scattering at very low-q (i.e., less than about 0.02 Å^{-1}) is mainly due to air bubbles (see Fig. S5) and differs slightly from sample to sample, the subtraction is not meaningful in this q range.

(Figure continued next page.)

B. With precipitant addition

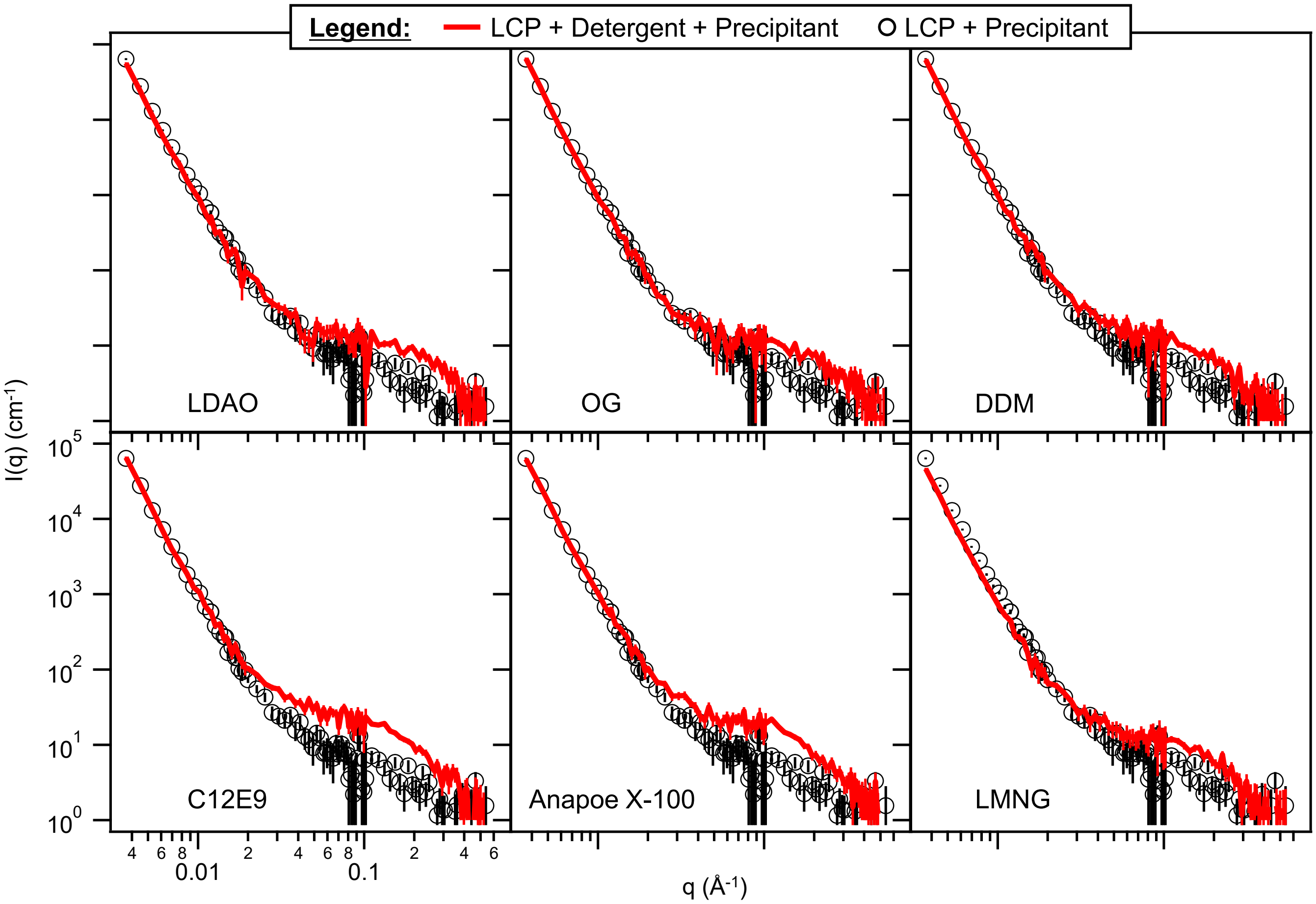

C. LCP without detergent

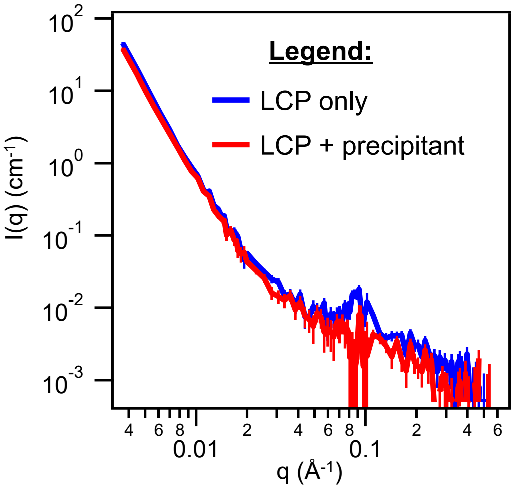

(Figure S4 – continued) **(B)** Subtraction of the LCP + precipitant curve (black circles) from the LCP + detergent + precipitant curves (solid red lines), and division by the detergent concentration in mg/mL, yields the red curves in Fig. 2. **(C)** Scattering from LCP alone (no detergent) without and with 2 M Na/K phosphate precipitant (same data as black triangles and circles in **A** and **B** respectively).

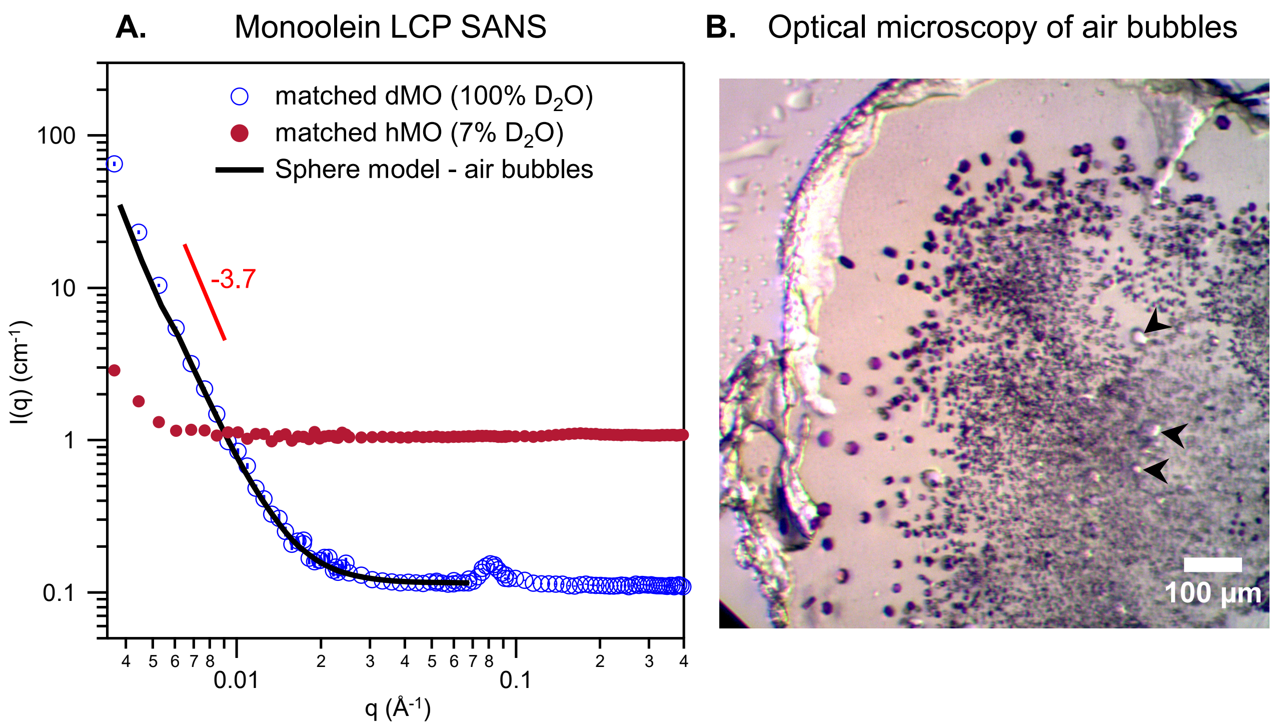

Fig. S5 – Effect of small air bubbles on the low-q scattering. Although the low-q scattering (q ≲ 0.02 Å^{-1}) was not needed for our conclusions, we have analyzed it in order to fully understand the sample, and have concluded that it was due to air bubbles. Because of the small volumes (usually around 25 µL each of lipid and aqueous components) and manual syringe mixing process used to produce the LCP, it is very difficult to purge all air from the system prior to mixing. In addition, to prevent denaturation, protein solutions must be kept cold prior to mixing. This results in an increased solubility for dissolved air, which then precipitates from solution upon mixing of the LCP at room temperature. In D_2O, entrapped air bubbles result in a large scattering signal at very low-q (see **A**, blue circles, q ≲ 0.02 Å^{-1}). Degassing after mixing was always attempted, and did slightly reduce the low-q scattering compared to non-degassed samples, but did not effectively remove small bubbles from the viscous cubic phase. Several lines of evidence indicate that this low-q scattering is from air bubbles, and not some other unidentified source: (1) The low-q scattering of dMO LCP in 100% D_2O, near its match point, can be compared to that of hMO LCP at its match point of 7% D_2O. Because the SLD of 7% D_2O (-0.07×10^{-6} A^{-2}) is very near that of air (SLD of 0), any air in hMO LCP would be nearly contrast matched, and indeed the low-q scattering is greatly reduced under this condition (**A**, red curve). (2) The slope of the low-q power law is very near to -4.0, as would be expected for a smooth interface from air. (3) Because the data are on an absolute scale, it is also possible to fit a more detailed model to explain the observed scattering intensity. For instance, fitting a 20% polydisperse sphere model with an assumed volume fraction of 1%, a sphere SLD of 0 (for air), and solvent SLD of 6.38×10^{-6} Å^{-2} (for D_2O) gives a bubble diameter of about 22 µm, consistent with air bubbles often seen when LCP is examined by optical microscopy (see **B**, arrows). The bubble radius and volume fraction are completely correlated as fit parameters; at a given intensity, smaller volume fractions of air imply smaller bubbles. We do not believe more than 2% air could have been introduced during mixing, based on microscopy. Fortunately, scattering from air is well-separated from the signals of interest from bR and detergent micelles, which is in the Q range of 0.02–0.2 Å^{-1}. Error bars are plus or minus one standard deviation and represent the uncertainty due to counting statistics.

Compound/ Fragment	Formula in H2O	Estimated Vol. (Å^3)	SLD ($\times 10^{-6}$ Å^2)	Formula after D2O exchange	SLD (×10-6 Å^2)
hOG tail	C8H17	218.8	-0.48	C8H17	-0.48
hMO tail	C17H33	442.5	-0.24	C17H33	-0.24
LDAO	C14H31NO	414.9	-0.19	C14H31NO	-0.19
FC10	C15H34NO4P	525.5	0.19	C15H34NO4P	0.19
C13E8	C29H60O9	879.3	0.23	C29H59D1O9	0.35
C12E8	C28H58O9	853.8	0.25	C28H57D1O9	0.37
C10E6	C22H46O7	674.7	0.22	C22H45D1O7	0.37
C12E9	C30H62O10	918.3	0.28	C30H61D1O10	0.39
C12E10	C32H66O11	985.0	0.30	C32H65D1O11	0.41
C10E9	C28H58O10	918.9	0.30	C28H57D1O10	0.41
A58	C56H114O21	1728.7	0.39	C56H113D1O21	0.45
A35	C58H118O24	1816.1	0.46	C58H117D1O24	0.52
hMO	C21H40O4	620.4	0.21	C21H38D2O4	0.55
AX-305	C74H142O31	2260.0	0.62	C74H141D1O31	0.67
AX-114	C30H54O9	841.5	0.57	C30H53D1O9	0.69
TX-100	C34H62O11	972.0	0.60	C34H61D1O11	0.70
NP-40	C32H58O10	905.8	0.59	C32H57D1O10	0.71
A20	C58H114O26	1935.2	0.57	C58H111D3O26	0.73
A80	C64H124O26	1949.0	0.58	C64H121D3O26	0.74
DDM	C24H46O11	719.9	0.71	C24H39D1O11	1.20
hOG	C14H28O6	465.1	0.50	C14H24D4O6	1.39
C14M	C26H50O11	765.2	0.65	C26H43D7O11	1.60
OGNG	C27H52O12	857.9	0.64	C27H44D8O12	1.61
DM	C22H42O11	722.2	0.73	C22H35D7O11	1.74
C13M	C25H48O11	741.2	0.68	C25H41D8O11	1.75
Cymal 5	C23H42O11	724.5	0.82	C23H35D7O11	1.83
LMNG	C47H88O22	1367.1	0.81	C47H74D14O22	1.88
hMO hg	C4H7O4	177.9	1.33	C4H5D2O4	2.50
hOG hg	C6H11O6	246.3	1.36	C6H7D4O6	3.05
dtOG	C14H11D17O6	465.1	4.30	C14H7D21O6	5.20
dMO hg	C4H(2.1)D(4.9)O4	177.9	4.20	C4H(0.1)D(6.9)O4	5.37
dOG hg	C6H4D7O6	246.3	4.32	C6D11O6	6.01
dMO	C21H(4.6)D(35.4)O4	620.4	6.16	C21H(2.6)D(37.4)O4	6.49
dOG	C14D24H4O6	465.1	5.87	C14D28O6	6.77
dMO tail	C17H(2.5)D(30.5)	442.5	6.94	C17H(2.5)D(30.5)	6.94
dOG tail	C8D17	218.8	7.62	C8D17	7.62

Table S2 – Calculated SLDs of detergents, lipids and fragments. The table is grouped into classes: H-tail fragment, zwitterionic, alkyl PEG, monoolein, octylphenol-PEG, polysorbates, alkyl glycosides, headgroups, and (in light blue) deuterated fragments. Calculated SLDs should be taken as approximate, as they depend on the solvated molecular volumes, which were estimated using the software Vega ZZ and have not been experimentally measured. Abbreviations: dt – tail-deuterated; d – fully-deuterated; h – non-deuterated; hg – headgroup.

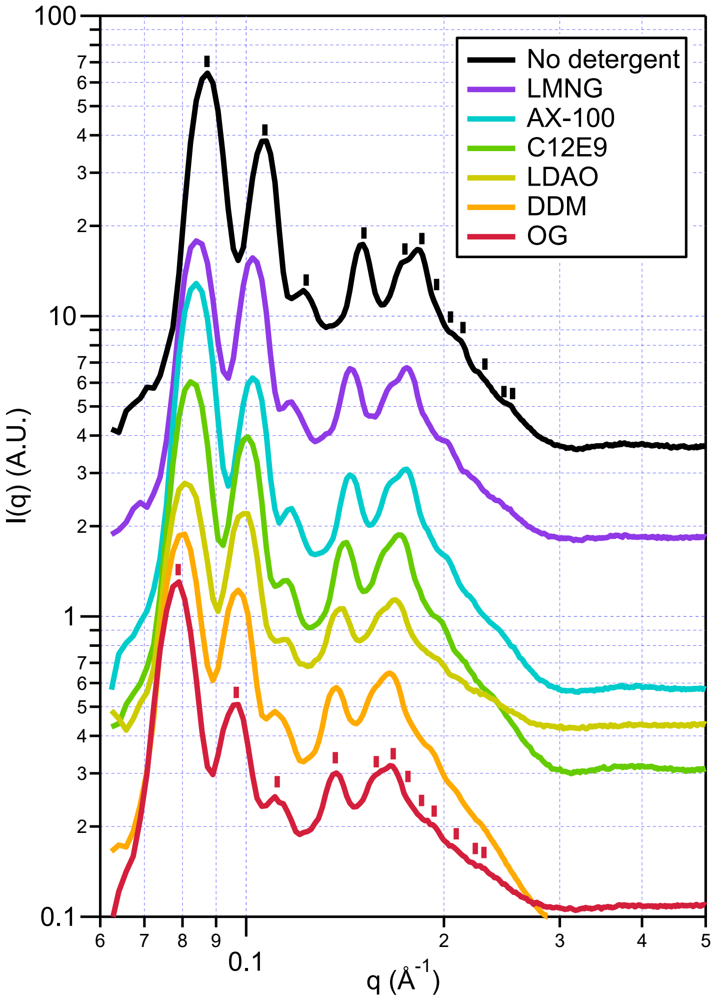

Detergent	Lattice Parameter (Å)
OG	113.8
DDM	111.8
LDAO	108.1
C12E9	108.1
TX100	106.4
LMNG	106.4
No detergent	101.4

Figure S6 – Indexing of LCP/detergent mixtures by SAXS. Curves are vertically offset for clarity. The lattice parameters are tabulated for the different mixtures. The same samples were used as for SANS (Fig. 2), but the Bragg peaks are largely absent in SANS due to contrast matching. Intensities have not been put on an absolute scale. Expected peak positions for the Pn3m cubic phase are marked for the two extremes ("No detergent" and "OG").

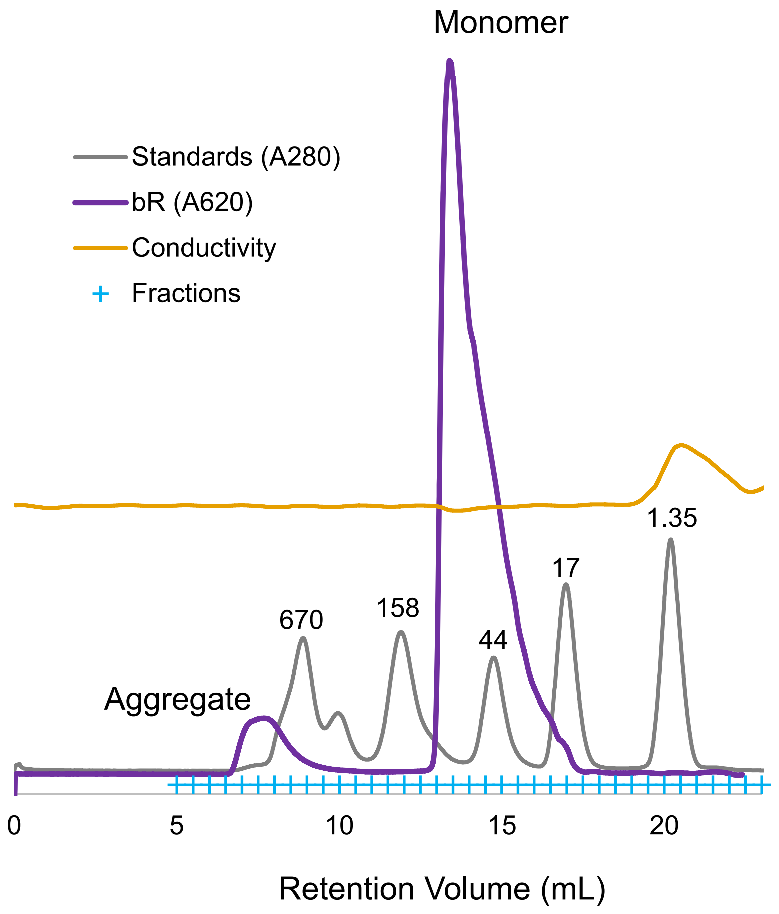

Fig. S7 – SEC purification of bR. After solubilization in OG, SEC was used to separate monomeric bR from aggregates. Peak fractions were collected and concentrated for incorporation into LCP. Protein was never frozen after performing SEC. Because bR was highly concentrated, absorbance was monitored at 620 nm (away from the bR absorption maximum at 550 nm) in order to remain in the linear range of the detector. Standards were used to determine the apparent molecular weight of the monomeric bR/OG complex, which was found to be 71.2 kDa. Given the expected bR monomer mass of 27.2 kDa, this implies roughly 44 kDa of bound OG, consistent with a published measurement of 35 ± 10 kDa (Santonicola, M.G., Lenhoff, A.M., Kaler, E.W., 2008. Biophysical Journal 94(9):3647-3658).

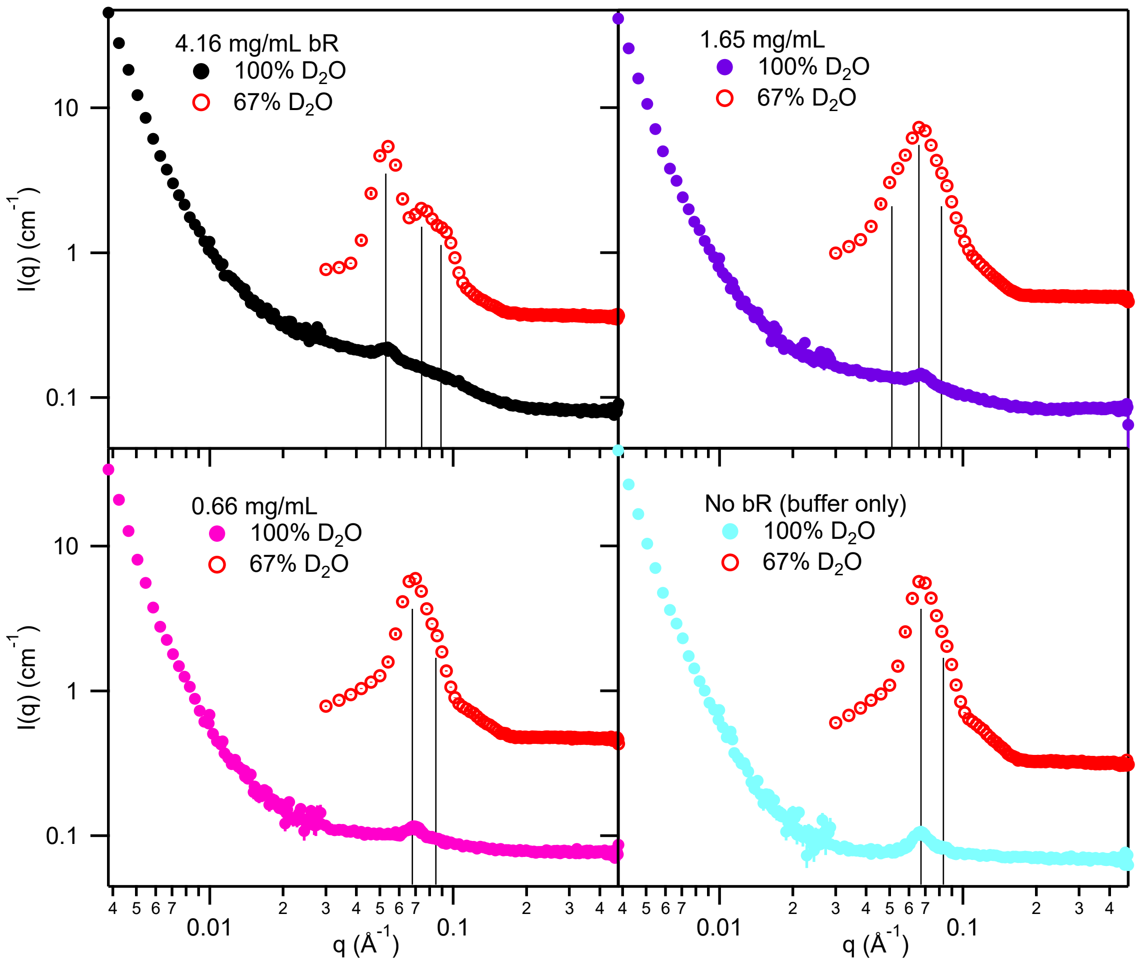

Figure S8 – SANS from bR/cmOG/LCP away from the LCP match point. The samples from Fig. 3A were brought away from the LCP match point (near 100% D_2O) by removing 1/3 of the D_2O buffer from the cuvette, and replacement with H_2O buffer, resulting in a solvent containing 67% D_2O. This allowed for the determination of which Bragg peaks were due to the lipidic matrix, since these would greatly increase in intensity away from the LCP match point. Conversely, this brought the solvent nearer to the match point of the protein (about 42% D_2O). Therefore, if any Bragg peaks originated from ordering of the protein, these would decrease in intensity. All of the residual Bragg peaks seen near the LCP match condition were found to increase greatly in intensity when H2O was added, indicating an origin in lipid structures rather than protein. Peak positions in samples with no bR or 0.66 mg/mL bR were consistent with the Pn3m phase (ratios of the q positions of the first peak to its shoulder, marked with vertical lines, were near $\sqrt{2}:\sqrt{3}$). At the highest bR concentration of 4.16 mg/mL, a new peak appears at $q \approx 0.053$ Å^{-1}, indicating a phase change, although this could not be confidently indexed due to the poor q-resolution and small number of Bragg peaks. The onset of this behavior can also be seen in an additional shoulder near $q \approx 0.05$ Å^{-1} in the scattering from the 1.65 mg/mL bR sample.

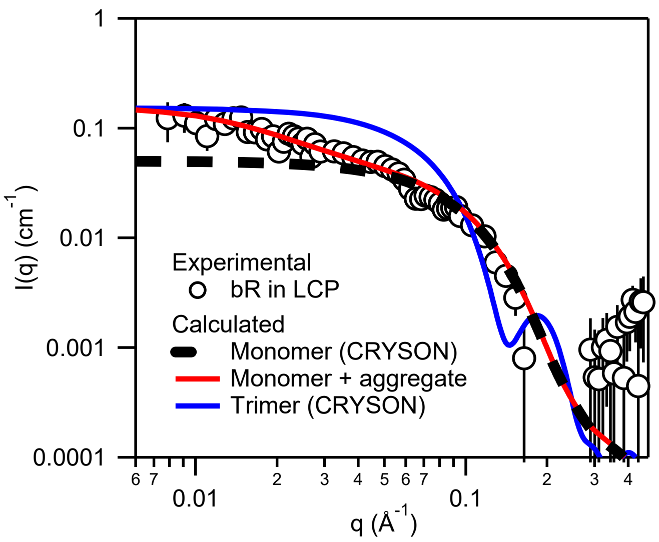

Fractal aggregate model fit

Parameter	Fit Value	
Scale	1	(fixed)
Background	0	(fixed)
Volume Fraction	$(4.9 \pm 0.3)\times10^{-5}$	
Radius (nm)	1.88	(fixed)
Fractal Dimension	3	(fixed)
Correlation Length (nm)	4.2 ± 0.2	
SLD block ($\times10^{-6}$ Å^{-2})	2.91	(fixed)
SLD solvent ($\times10^{-6}$ Å^{-2})	6.4	(fixed)

Figure S9 – Comparison of bR/LCP scattering to theoretical scattering from bR monomer and trimer. The experimental scattering curve from 1.65 mg/mL bR in LCP is shown, after subtraction of the residual LCP scattering (as in Fig. 3B). CRYSON was used to compute theoretical scattering curves from atomistic models. The bR monomer and trimer structures used in the calculation were from PDB code 1c3w with lipids deleted. Calculated scattering from CRYSON was not scaled to the experimental data, but was placed on an absolute scale using I_0 values from the SASSIE Contrast Calculator; thus, the good agreement between the calculated and experimental scattering intensity is notable. The experimental bR/LCP scattering was consistent with the calculated monomeric scattering plus a small amount of fractal aggregate, as determined by fitting using SasView with the built-in "fractal" model (see Fig. S2 for additional details on SasView fitting procedures). The volume fraction of aggregate was 4.91×10^{-5}, which corresponds to 0.066 mg/mL bR, or 4.0% of the total protein mass, present as aggregate. In contrast to the monomeric case, the calculated scattering for trimeric bR was inconsistent with the experimental data.

Detergent	Abbreviation	Supplier	Product Number
Fos-Choline 10	FC10	Anatrace	F304 1 GM
Lauryldimethylamine Oxide	LDAO	Anatrace	D360 1 GM
Decyl Maltoside	DM	Anatrace	D322 1 GM
Cymal-5	Cymal-5	Anatrace	C325 1 GM
Anapoe-C10E9	C10E9	Anatrace	DSOL-ANP10
Anapoe-C10E6	C10E6	Anatrace	DSOL-ANP10
Dodecyl Maltoside	DDM	Anatrace	D399-M1216 1 KT
Anapoe-C12E10	C12E10	Anatrace	DSOL-ANP10
Octyl Glucoside	OG	Anatrace	O311 25 GM
Anapoe-C13E8	C13E8	Anatrace	DSOL-ANP10
Octyl Glucose Neopentyl Glycol	OGNG	Anatrace	NG311 1 GM
Anapoe-C12E9	C12E9	Anatrace	DSOL-ANP10
Anapoe-C12E8	C12E8	Anatrace	DSOL-ANP10
Anapoe X-305	AX-305	Anatrace	DSOL-ANP10
Anapoe 35	A35	Anatrace	DSOL-ANP10
Anapoe 20	A20	Anatrace	DSOL-ANP10
Tridecyl Maltoside	C13M	Anatrace	D399-M1216 1 KT
Anapoe 58	A58	Anatrace	DSOL-ANP10
Triton X-100 (SurfactAmps)	SX-100	Pierce	28314
Elugent	Elugent	EMD Millipore	324707-100ML
Anapoe-NID-P40	NP40	Anatrace	DSOL-ANP10
Anapoe X-100	AX-100	Anatrace	DSOL-ANP10
Anapoe 80	A80	Anatrace	DSOL-ANP10
Tetradecyl Maltoside	C14M	Anatrace	D399-M1216 1 KT
Lauryl Maltose Neopentyl Glycol	LMNG	Anatrace	NG310 1 GM
Anapoe X-114	AX-114	Anatrace	DSOL-ANP10
Tail-Deuterated Octyl Glucoside	dtOG	Anatrace	O311T
Fully Deuterated Octyl Glucoside	dOG	Anatrace	O311D
Monoolein	MO	Nu-Chek Prep	M-239
Deuterated Monoolein	dMO	*	

Table S3 – Detergent and lipid sources and product numbers. *Deuterated monoolein prepared by the National Deuteration Facility, Australian Nuclear Science and Technology Organisation.

Detergents were not chosen or compared for purity or suitability between different suppliers. These products are identified only in order to specify adequately the experimental procedure. In no case does such identification imply recommendation or endorsement by the National Institute of Standards and Technology, nor does it imply that the products are necessarily the best for the purpose.

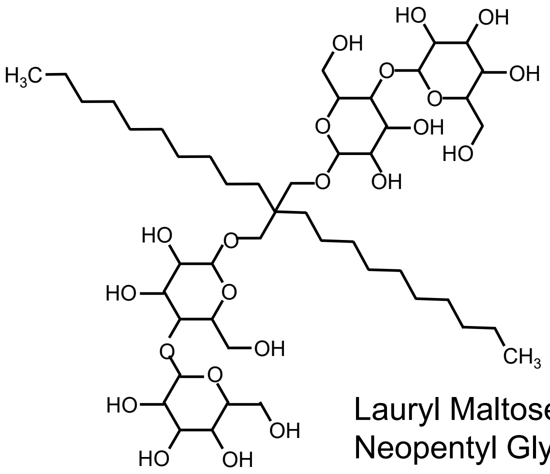

Lauryl Maltose
Neopentyl Glycol

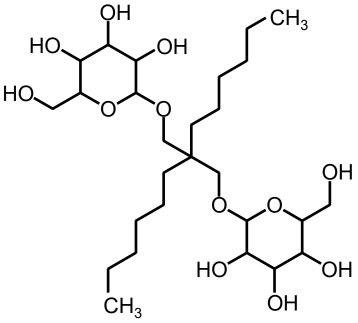

Octyl Glucose
Neopentyl Glycol

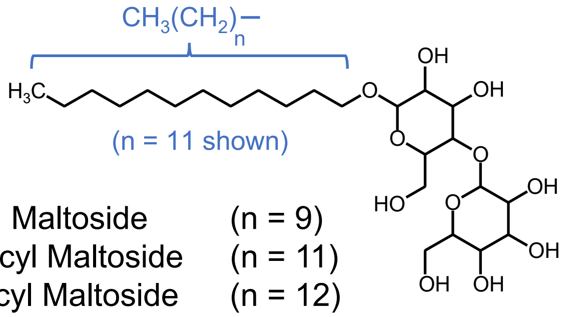

Decyl Maltoside	(n = 9)
Dodecyl Maltoside	(n = 11)
Tridecyl Maltoside	(n = 12)
Tetradecyl Maltoside	(n = 13)

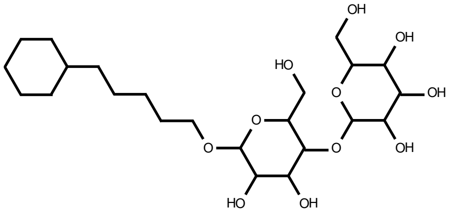

Cymal-5

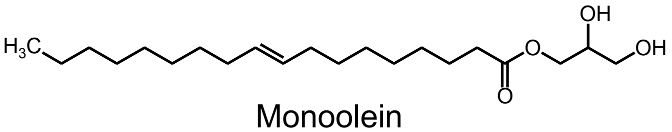

Monoolein

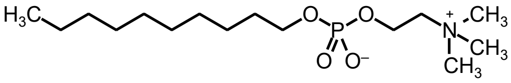

Fos-Choline 10

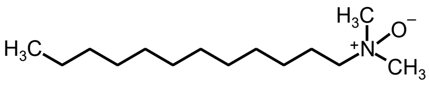

Lauryldimethylamine Oxide

Fig. S10 - Detergent and Lipid Structures

(Figure continued next page)

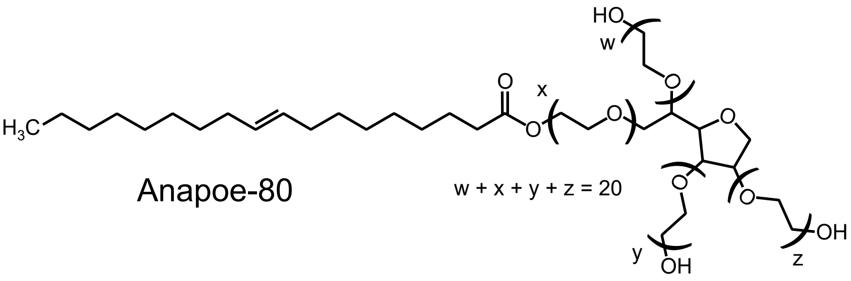

Anapoe-80

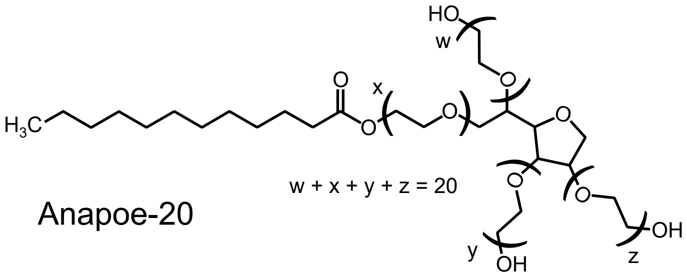

Anapoe-20

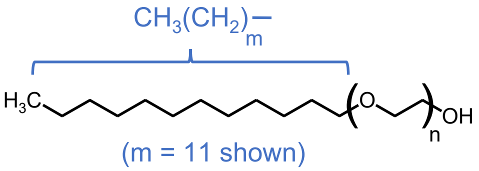

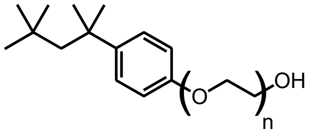

C10E9	(m = 9, n = 9)
C10E6	(m = 9, n = 6)
C12E8	(m = 11, n = 8)
C12E9	(m = 11, n = 9)
C12E10	(m = 11, n = 10)
Anapoe 35	(m = 11, n ≈ 23)
C13E8	(m = 12, n = 8)
Anapoe 58	(m = 15, n ≈ 20)

Anapoe X-114	(n = 7-8)
Anapoe NID-P40	(n = 8.5-9.5)
Triton X-100	(n = 9-10)
Anapoe X-305	(n ≈ 30)

(Figure S10 – continued from previous page)

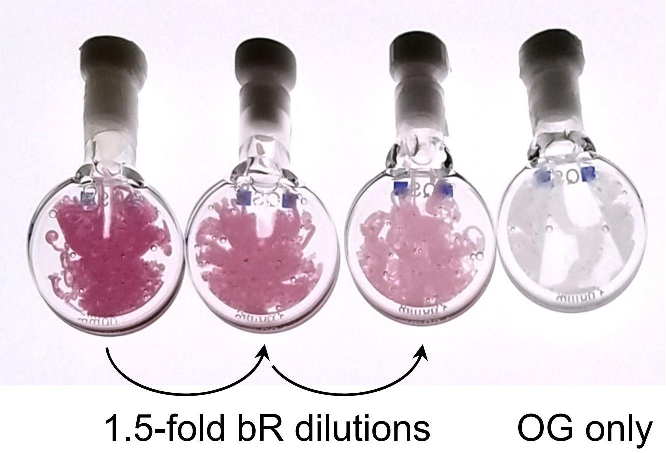

Figure S11 – Insertion of LCP into cuvettes. Single-phase LCP mixtures are difficult to prepare consistently, since the LCP has a narrow range where the Pn3m phase is formed (near full hydration) and the location of the phase boundary is sensitive with respect to conditions such as temperature, salt, and the amount of detergent added. Instead, we prepare two-phase dispersions of LCP in an excess of buffer. This ensures that the LCP is always exactly at full hydration. Since the buffer and LCP are near the contrast-matched condition, scattering from the LCP grain/solution interfaces is minimal. Dispersion of the LCP also facilitates degassing after insertion into cuvettes, which is important for reducing the large amount of low-q scattering often present from small air bubbles.

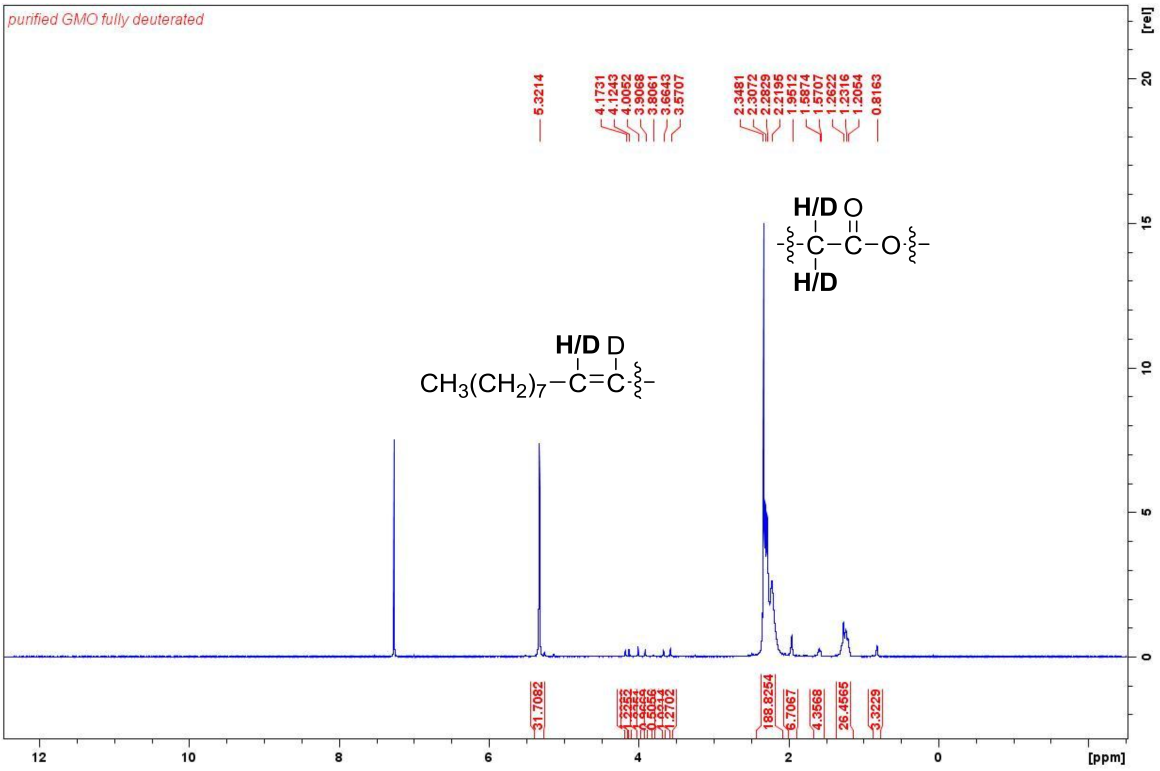

Figure 1. ^{1}H NMR spectrum of Monoolein-d$_{38}$ (CDCl$_3$, 400 MHz). Back exchange (D/H) occurs at the labelled sites.

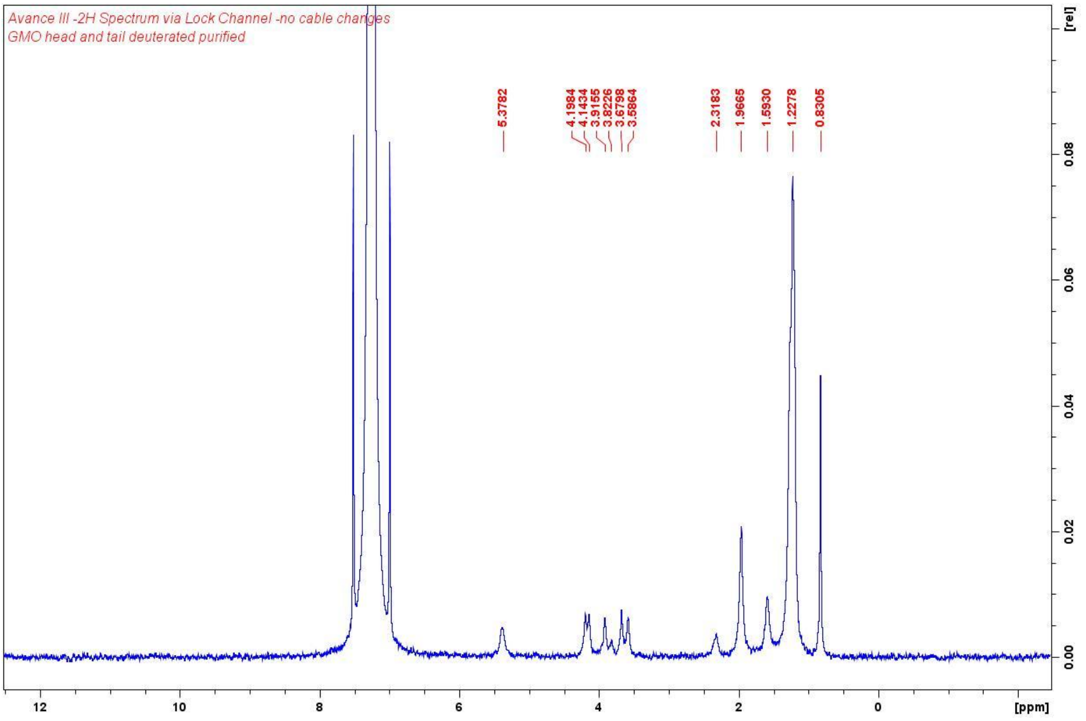

Figure 2. ^{2}H NMR spectrum of Monoolein-d$_{38}$ (CDCl$_3$, 61.4 MHz).

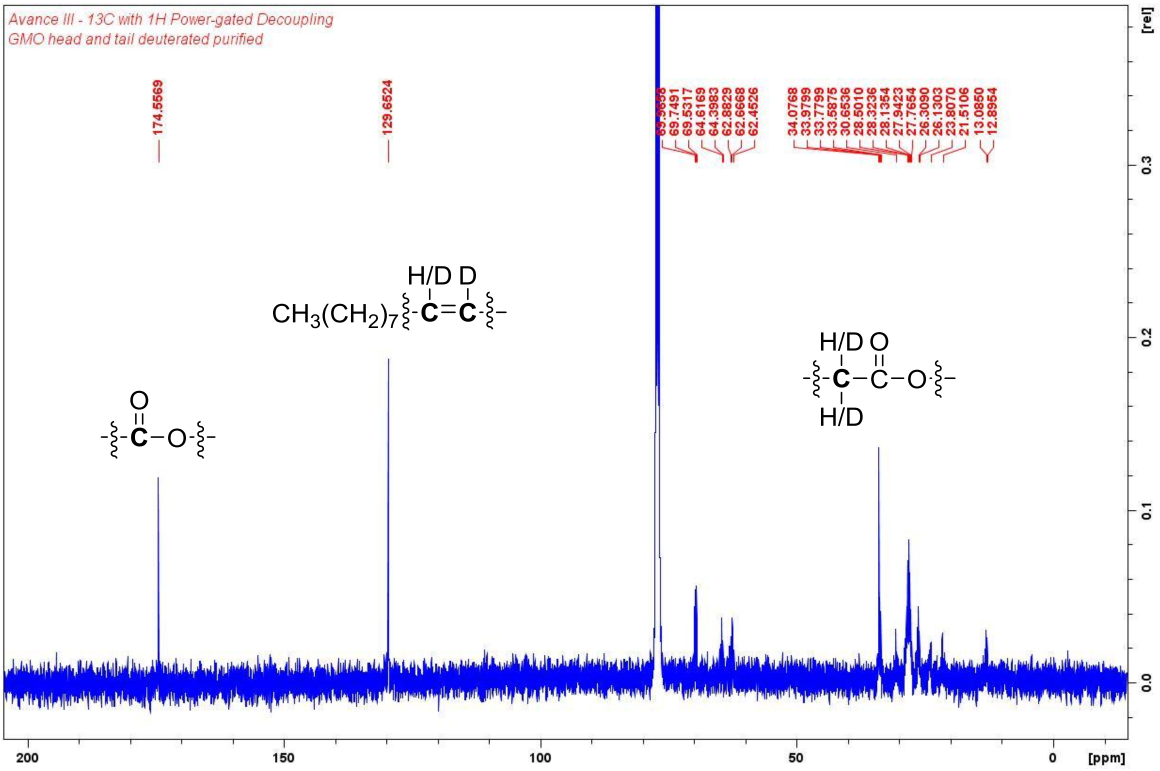

Figure 3. ^{13}C (^{1}H decoupled) NMR spectrum of Monoolein-d_{38} ($CDCl_3$, 100 MHz).

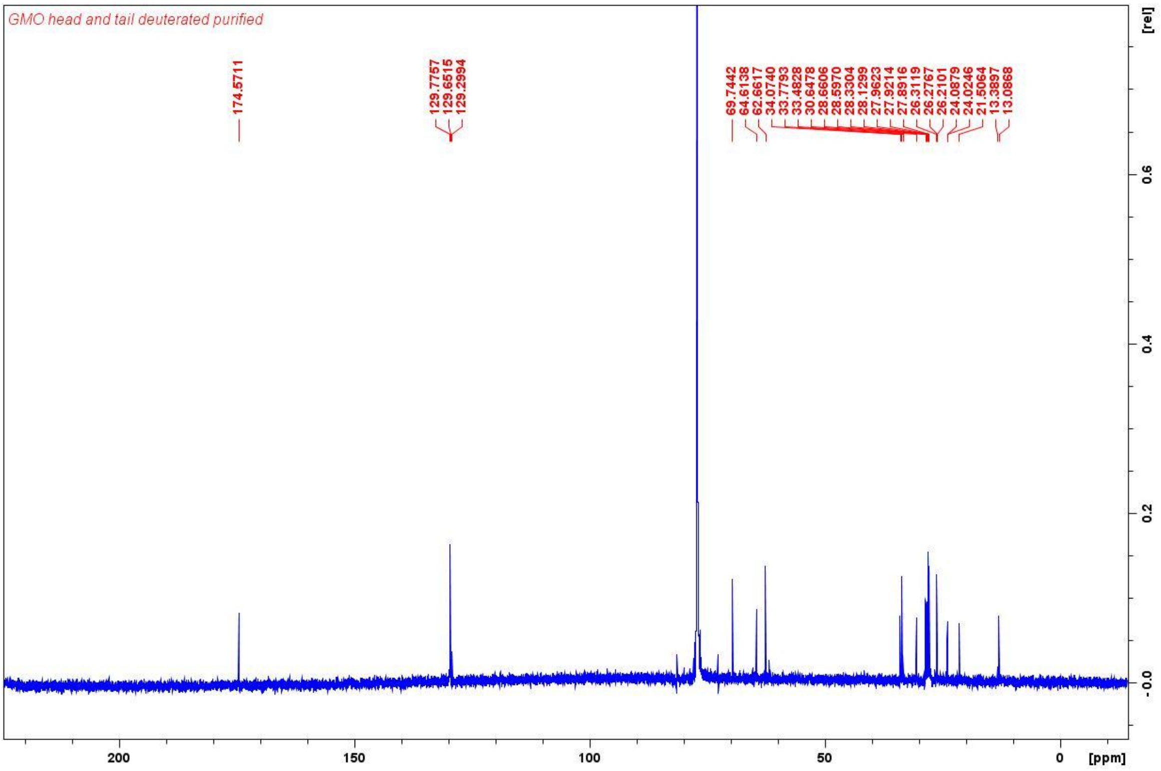

Figure 4. ^{13}C (^{1}H and ^{2}H decoupled) NMR spectrum of Monoolein-d_{38} ($CDCl_3$, 100 MHz).

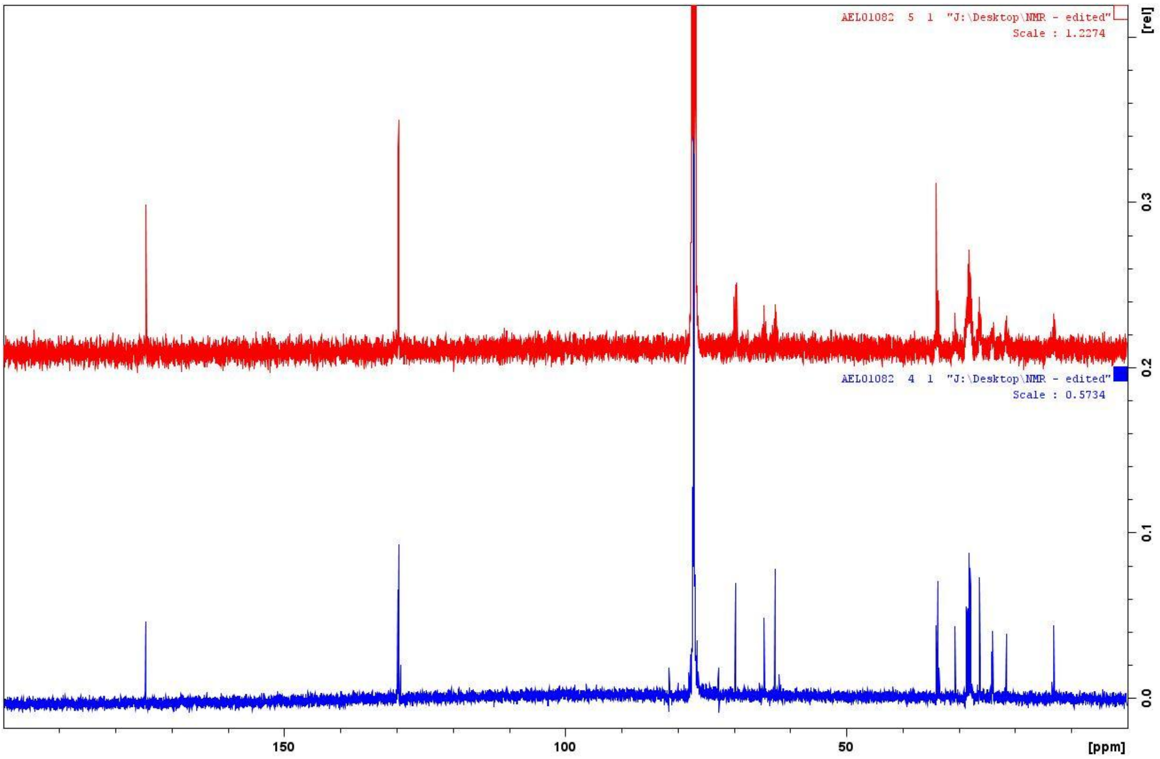

Figure 5. Comparison of ^{13}C (^{1}H decoupled) (top) and ^{13}C (^{1}H and ^{2}H decoupled) (bottom) NMR spectra of Monoolein-d_{38} ($CDCl_3$, 100 MHz).

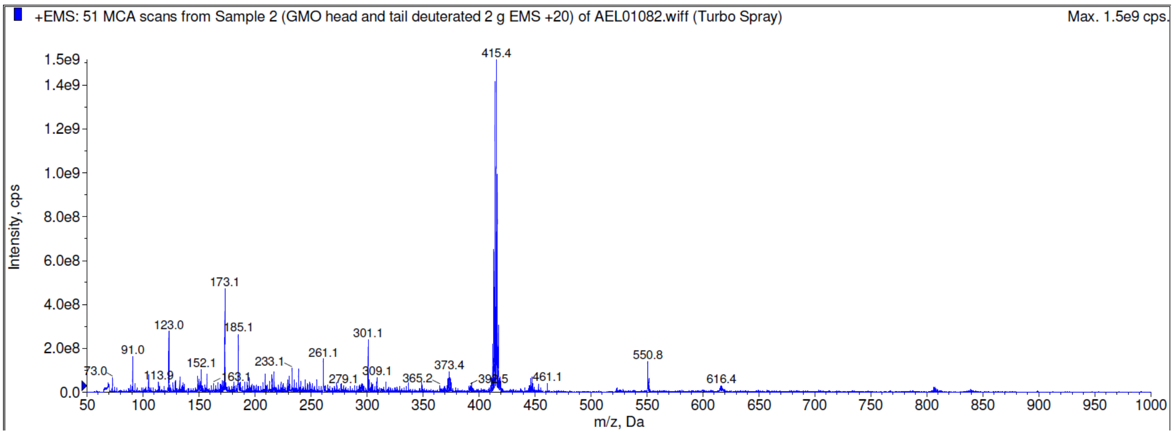

Figure 6. Mass spectrum of Monoolein-d_{38} (ESI+) $[M+Na]^+$.

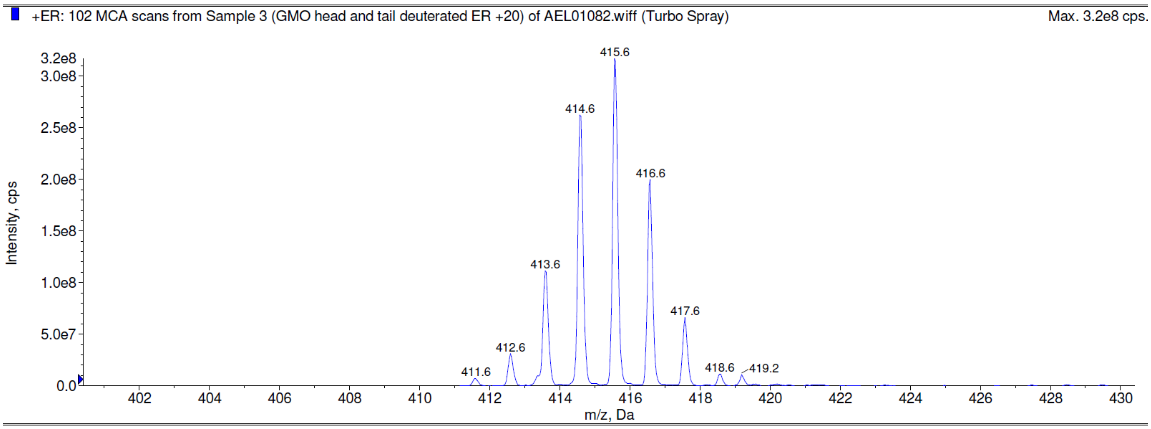

Peak List for " +ER: 102 MCA scans from Sample 3 (GMO head and tail deuterated ER +20) of AEL01082.wiff (Turbo Spray)"

	m/z (Da)	Resolution	Intensity (cps)	Peak area (counts)	Charges	Width (Da)	% Intensity
1	411.6000	2388.6523	6.9833e6	4.3333e7	1	0.1723	2.2266
2	412.5900	2577.0179	3.1125e7	1.8098e8	Undefined	0.1601	9.6910
3	413.5800	2488.2031	1.1155e8	6.7981e8	1	0.1662	35.3123
4	414.5700	2539.7947	2.6238e8	1.5689e9	1	0.1632	82.9295
5	415.5600	2626.7659	3.1717e8	1.8442e9	Undefined	0.1582	100.0000
6	416.5800	2713.1571	1.9998e8	1.1328e9	1	0.1535	63.0005
7	417.5700	2761.0988	6.6300e7	3.6658e8	1	0.1512	20.6420
8	418.5600	2765.8467	1.1542e7	6.6508e7	1	0.1513	3.6959
9	419.1900	3165.0976	1.0942e7	5.8725e7	Undefined	0.1324	3.2375

Figure 7. Mass spectrum of Monoolein-d_{38} (ER+) $[M+Na]^+$.

Calculation of % Deuteration from MS Isotope Distribution

Note: Always label the lowest m/z peak as Peak No. 1 & increment each subsequent peak by 1 m/z unit

Parent Ion Peak No.	Theoretical % D for Peak	Peak Area	Corrected Peak Area	Contribution of Peak to Overall % D	Theoretical % D for Peak	Corrected Peak Area	Amount (%) of compound at Theoretical % D
1	84.2	4.3333E+07	4.3333E+07	0.8	84.2	4.3333E+07	1.0
2	86.8	1.8098E+08	1.7114E+08	3.3	86.8	1.7114E+08	3.9
3	89.5	6.7981E+08	6.3730E+08	12.9	89.5	6.3730E+08	14.4
4	92.1	1.5689E+09	1.4086E+09	29.2	92.1	1.4086E+09	31.7
5	94.7	1.8442E+09	1.4656E+09	31.3	94.7	1.4656E+09	33.0
6	97.4	1.1328E+09	6.6239E+08	14.5	97.4	6.6239E+08	14.9
7	100.0	3.6658E+08	4.8576E+07	1.1	100.0	4.8576E+07	1.1
8			0.0000E+00	0.0	0.0	0.0000E+00	0.0
9			0.0000E+00	0.0	0.0	0.0000E+00	0.0
10			0.0000E+00	0.0	0.0	0.0000E+00	0.0
11			0.0000E+00	0.0	0.0	0.0000E+00	0.0
12			0.0000E+00	0.0	0.0	0.0000E+00	0.0
13			0.0000E+00	0.0	0.0	0.0000E+00	0.0
14			0.0000E+00	0.0	0.0	0.0000E+00	0.0
15			0.0000E+00	0.0	0.0	0.0000E+00	0.0
16			0.0000E+00	0.0	0.0	0.0000E+00	0.0
17			0.0000E+00	0.0	0.0	0.0000E+00	0.0
18			0.0000E+00	0.0	0.0	0.0000E+00	0.0
19			0.0000E+00	0.0	0.0	0.0000E+00	0.0
20			0.0000E+00	0.0	0.0	0.0000E+00	0.0
21			0.0000E+00	0.0	0.0	0.0000E+00	0.0
22			0.0000E+00	0.0	0.0	0.0000E+00	0.0
23			0.0000E+00	0.0	0.0	0.0000E+00	0.0
24			0.0000E+00	0.0	0.0	0.0000E+00	0.0
25			0.0000E+00	0.0	0.0	0.0000E+00	0.0
26			0.0000E+00	0.0	0.0	0.0000E+00	0.0
27			0.0000E+00	0.0	0.0	0.0000E+00	0.0
28			0.0000E+00	0.0	0.0	0.0000E+00	0.0
29			0.0000E+00	0.0	0.0	0.0000E+00	0.0
30			0.0000E+00	0.0	0.0	0.0000E+00	0.0

^{13}C Isotopic Distribution from ChemDraw:	
^{13}C # 2	22.7
^{13}C # 3	3.3

Total Corrected Area of all Isotopic Peaks
4.4370E+09

Average % Deuteration of

93.2

Error = +/- 2 %

(yellow)	Input Data
(green)	Calculated

Figure 8. Overall deuteration calculation by mass spectrometry of Monoolein-d_{38}.

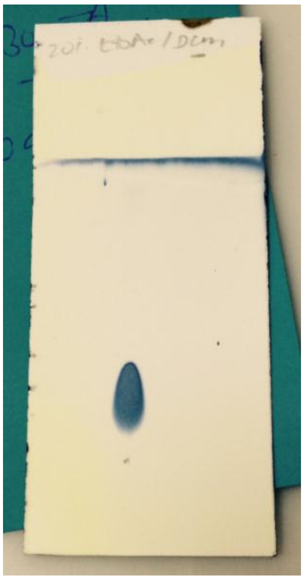

Figure 9. Silica gel TLC plate of Monoolein-d_{38} (20% EtOAc in DCM; visualised with Hanessian's TLC stain (ceric ammonium molybdate).

1. Glycerol-d_8 (98% deuterated) was purchased from Sigma-Aldrich; it is assumed that no H/D exchange would occur during the subsequent synthesis.
2. The percentage deuteration of the tail was calculated assuming that the deuteration of the head remained as 98% and having determined the overall deuteration of the molecule to be 93.2% (±2%) via mass spectrometry:
$(0.98 \times 5) + (33 \times x) = (0.932 \times 38)$
$x = (0.932 \times 38) - (0.98 \times 5) / 33$
$x = 92.5\%$
Back exchange (D/H) occurs at the positions indicated below:

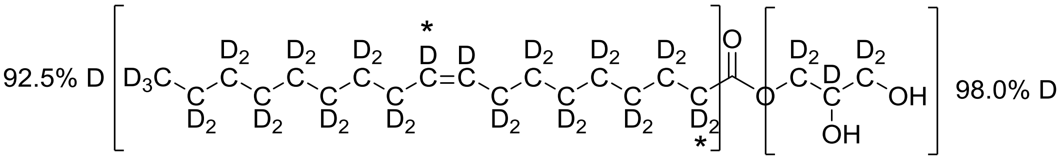

Figure 10. Deuterated Monoolein-d_{38} showing the percentage deuteration in the head and tail. *Sites which have undergone back (D/H) exchange.

3. Sigma-Aldrich product information sheet for 1-Oleoyl-*rac*-glycerol and the references within.
4. Mattson, F. H. and Volpenhein, R. A. *J. Lipid Research* **1962**, 3, *3*, 281-296.
