# Supplementary material for: Direct localization of detergents and bacteriorhodopsin in the lipidic cubic phase by small-angle neutron scattering
Source: IUCrJ. 2021 Jan 1;8(Pt 1):22–32. doi: 10.1107/S2052252520013974 (PMC7792994; doi:10.1107/S2052252520013974)
Supplement: Supplementary file 2 [file m-08-00022-sup2.pdf]

Australian Government

ansto
Nuclear-based science benefiting all Australians

THE NATIONAL DEUTERATION FACILITY

PRODUCT ANALYSIS CERTIFICATE

Product name: Monoolein-d_{38} (1-Oleoyl-*rac*-glycerol-d_{38})

Lab book code: AEL01078 and AEL01082 (NMR experiments: AEL01078 expt 1; AEL01082 expt 2, 3 and 4)

Product structure:

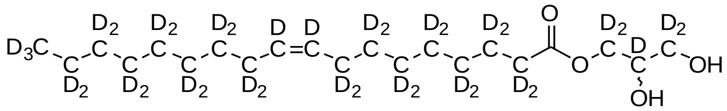

Proposal & round: DD4340 (2016-1)

Batch number: CoA0915

Quantity supplied: Quantity: 2.1 g

Supplied to: Paul Butler and Thomas Cleveland

Date synthesised: 20/04/2015

Date tested: 24/04/2015

Product Information:

Theoretical chemical formula: $C_{21}H_{2}D_{38}O_{4}$

Theoretical molecular weight: 394.53 g/mol (if 100%D)

Physical appearance: White solid at 0 °C

Storage conditions: Store below 0 °C; store under inert gas; do not store in solution.

Stability: Stable under the recommended conditions.

Chemical purity: Pure* by NMR and TLC (TLC attached).

Isotopic purity: 93.2% (±2%) overall (determined by MS analysis); 98.0% in the head;[1] 92.5% (±2%) in the tail.[2]

The following spectra are attached:

NMR: ■ ^{1}H ■ ^{2}H ■ ^{13}C □ Other (please specify)

MS: ■

FTIR: □

Other: □ (Please Specify) ………

Comments: *Contains a small amount of 2-oleoyl-glycerol-d_{38} (acyl migration occurs upon storage or in solution);[3] typically exists as a mixture of 88:12 of 1- to 2-substituted lipids when stored in solution for long periods of times.[4]

Product synthesised by: Dr Anna Leung

NDF Approval by: Dr Tamim Darwish
